# Integrated O_2_ and H_2_ Gas Therapy via Microneedle‐Assisted Photocatalytic Water Splitting for Accelerating Diabetic Full‐Thickness Skin Wound Healing

**DOI:** 10.1002/advs.77413

**Published:** 2026-08-29

**Authors:** Zesheng Chen, Yanchao Yu, Xingyuan Xiao, Wenwen Cheng, Ye Yang, Zhengyao Zhang, Wang Wang, Zhijun Zhou, Weikang Hu, Xiaolong Wang, Bing Li, Kwang Leong Choy, Yun Chen, Zijian Wang

**Affiliations:** ^1^ Department of Urology Hubei Key Laboratory of Urological Diseases Institute of Urology Zhongnan Hospital of Wuhan University Wuhan China; ^2^ Department of Biomedical Engineering TaiKang Medical School (School of Basic Medical Sciences) Wuhan University Wuhan China; ^3^ School of Materials Science and Engineering Stem Cells and Tissue Engineering Manufacture Center Hubei University Wuhan China; ^4^ Suzhou Key Laboratory of Advanced Sustainable Materials & Technologies Division of Natural and Applied Sciences Duke Kunshan University Kunshan China; ^5^ Department of Pathology and Laboratory Medicine Temple University Lewis Katz School of Medicine Philadelphia Pennsylvania USA

**Keywords:** ferroptosis, gas therapy, microneedle, photocatalysis, wound healing

## Abstract

Impaired wound healing in diabetes is closely associated with cellular ferroptosis. Microneedle‐assisted gas therapy exerts molecular regulation of ferroptosis, representing a promising approach for accelerating diabetic wound healing. To date, O_2_ and H_2_ gas therapies have consistently been conducted independently and have never been integrated into a single system. Whether and how their synergy might be realized remains a defining challenge. Herein, we propose a concept of integrated O_2_ and H_2_ gas therapy, demonstrated by fabricating a gas‐producing microneedle material. Platinum@MIL‐101(Fe)‐NH_2_@phosphotungstic acid (PMP) nanoparticles are synthesized as a visible light‐driven photocatalyst for H_2_ and O_2_ co‐evolution. The rational design of a spatially separated structure boosts their photocatalytic efficiency under physiological conditions. PMP nanoparticles are further incorporated with bilayer gelatin methacryloyl (GelMA)/PMP composite microneedles (denoted as GPM microneedles). The obtained GPM microneedles enable transdermal delivery of PMP nanoparticles for integrated O_2_ and H_2_ gas therapy, and demonstrate desirable biocompatibility and pro‐regenerative effects. The mechanism of integrated O_2_ and H_2_ gas therapy has been identified as a TNFAIP3/hnRNPA1 ubiquitination/ferroptosis signaling axis, establishing for the first time a direct link to ferroptosis. In conclusion, this study yields a transformative concept and a ferroptosis‐targeting microneedle material with well‐defined molecular mechanism for diabetic wound management.

## Graphical abstract

A concept of integrated O_2_ and H_2_ gas therapy is achieved using a gas‐producing material. PMP nanoparticles, fabricated from platinum, MIL‐101(Fe)‐NH_2_, and phosphotungstic acid, are integrated into bilayer gelatin methacryloyl microneedles for transdermal delivery. These microneedles enable integrated O_2_ and H_2_ gas therapy and promote wound healing via modulation of the TNFAIP3/hnRNPA1 ubiquitination/ferroptosis signaling axis.

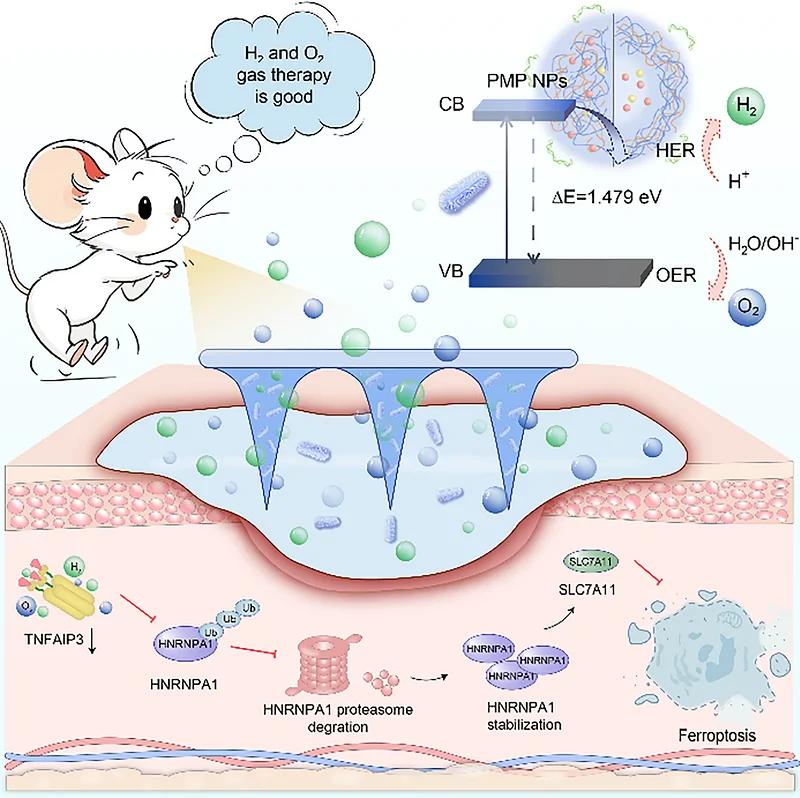

## 1 Introduction

Impaired wound healing in diabetes represents a global healthcare challenge, affecting over 18.6 million newly diagnosed patients annually [1]. Diabetic wounds are characterized by prolonged healing cycles and an increased risk of bacterial infection, which may ultimately lead to amputation or even death [2, 3]. Despite considerable research devoted to addressing this challenge, clinical treatment options for these wounds remain suboptimal. Ferroptosis is an iron‐dependent, non‐apoptotic form of regulated cell death (RCD) [4]. Jin et al. performed a single‐cell omics analysis on fresh skin tissues from diabetic patients and revealed excessive iron accumulation along with elevated ferroptosis levels in endothelial cells [5]. This phenomenon suggests that ferroptosis acts as a potential inducer of impaired wound healing in diabetes. Ferroptosis also plays a vital role in angiogenesis, oxidative stress injury, and energy metabolism [5, 6]. In recent years, researchers have developed a series of bioactive materials targeting ferroptosis to accelerate diabetic wound healing. For example, Zou et al. developed a baicalein‐laden supramolecular dressing that inhibits high glucose‐induced ferroptosis by modulating the Keap1/NRF2/HIF‐1α signaling axis [7]. Lin et al. reported an MMP‐9 responsive hydrogel that inhibits endothelial cell ferroptosis by activating transient receptor potential ankyrin 1 (TRPA1), thereby improving cell migration, proliferation, and tube formation [8]. These favorable results have validated ferroptosis as a promising therapeutic target.

Gas therapy mediated by oxygen (O_2_), hydrogen (H_2_), nitric oxide (NO), and hydrogen sulfide (H_2_S) is reshaping the paradigm of regenerative medicine, transitioning from macroscopic modulation to precise molecular signaling intervention [9, 10]. O_2_ and H_2_ gas therapies have attracted considerable attention among them, with a series of gas‐producing and/or gas‐delivering biomaterials reported. For example, Wu et al. fabricated a biocompatible microalgae‐gel patch that in situ produces hyperbaric O_2_ to promote diabetic wound healing [11]. He et al. prepared an H_2_‐incorporated titanium oxide nanorods for glucose depletion and hydrogen generation, which pioneered an era of H_2_ gas therapy [12]. Currently, the pro‐regenerative mechanisms of O_2_ gas therapy have been comprehensively investigated and summarized. These include, but are not limited to, enhanced survival and migration of keratinocytes and dermal fibroblasts, upregulation of angiogenic growth factors, and suppression of pro‐inflammatory cytokines [13]. Compared to O_2_ gas therapy, H_2_ gas therapy remains poorly understood. Wang et al. found that H_2_ gas therapy induces macrophage polarization from the M1 phenotype toward the M2 phenotype, marking a critical shift from destructive inflammation toward regenerative repair [14]. Our literature review indicates that studies on O_2_ and H_2_ gas therapies have consistently been conducted independently and have never been integrated into a single system. The synergistic effects of O_2_ and H_2_ gas therapies have long been overlooked, thus limiting their applications for diabetic wound healing.

**SCHEME 1:**
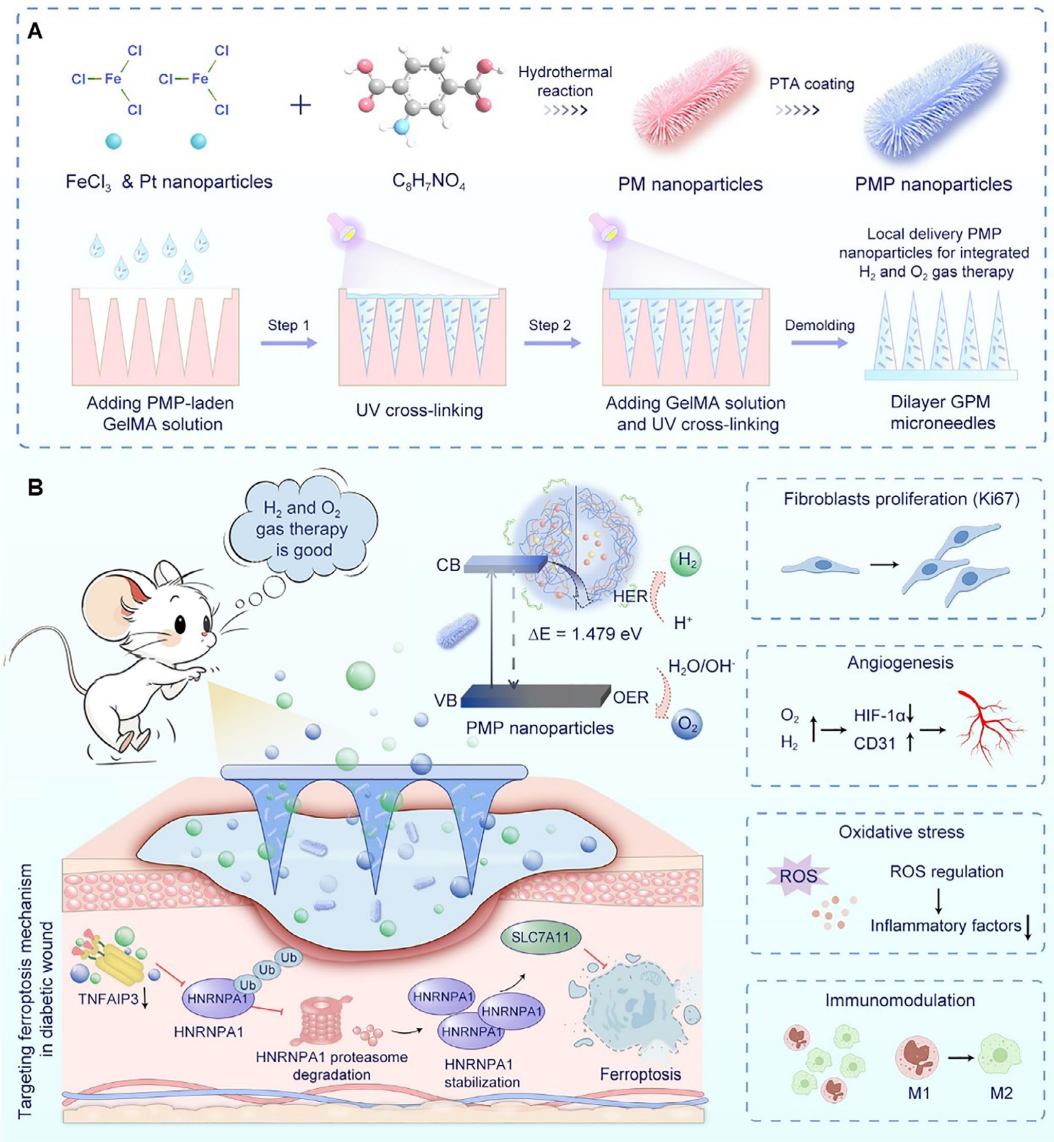
GPM microneedles and integrated O_2_ and H_2_ gas therapy for accelerating diabetic wound healing. (A) A diagram showing the preparation methods for GPM microneedles; (B) GPM microneedles accelerate diabetic wound healing by integrated O_2_ and H_2_ gas therapy. This study highlights both the spatially separated structure for enhanced O_2_ and H_2_ co‐evolution, and the molecular mechanisms of the TNFAIP3/hnRNPA1 ubiquitination/ferroptosis signaling axis underlying integrated O_2_ and H_2_ gas therapy.

To address the research gap, this study not only establishes a concept of integrated O_2_ and H_2_ gas therapy, but also fabricates a therapeutic platform for O_2_ and H_2_ co‐generation and co‐delivery. The preparation methods for the therapeutic platform are shown in Scheme 1A. First, platinum (Pt)‐incorporated MIL‐101(Fe)‐NH_2_ metal organic frameworks (namely PM nanoparticles) are synthesized using a hydrothermal reaction. Subsequently, PM nanoparticles are coated with a phosphotungstic acid (PTA) layer to obtain the composites (namely PMP nanoparticles). Featuring a spatially separated structure design, PMP nanoparticles efficiently generate O_2_ and H_2_ gases by photocatalytic overall water splitting. Compared to water electrolysis that requires an external electrical input for O_2_ and H_2_ co‐evolution, PMP nanoparticles utilize visible light energy instead of electrical input, rendering the gas co‐evolution reaction more biocompatible and sustainable [15]. Notably, the applications of PMP nanoparticles, as with most nanotherapeutic systems, are substantially constrained by inadequate delivery efficiency [16]. To solve this problem, PMP nanoparticles are further incorporated with biocompatible and biodegradable gelatin methacryloyl (GelMA) microneedles to obtain a bilayer composite material (namely GPM microneedles). By minimally invasive the skin tissue and precisely delivering PMP nanoparticles, GPM microneedles are assumed to enable O_2_ and H_2_ co‐generation and co‐delivery, which accelerates diabetic wound healing via integrated O_2_ and H_2_ gas therapy [17, 18].

This study further investigates the pro‐regenerative mechanisms of integrated O_2_ and H_2_ gas therapy, establishing for the first time a link to ferroptosis. In preliminary experiments, an RNA sequencing (RNA‐seq) has been performed on neo‐skin tissues. The results demonstrated significant enrichment of tumor necrosis factor alpha‐induced protein 3 (TNFAIP3)‐mediated ferroptosis, implicating this pathway in the pro‐regenerative effects of integrated O_2_ and H_2_ gas therapy. However, the downstream signals have yet to be elucidated. Scheme 1B shows a diagram of the proposed mechanisms. By downregulating the RNA expression of TNFAIP3, integrated O_2_ and H_2_ gas therapy inhibits the ubiquitination‐dependent degradation of heterogeneous nuclear ribonucleoprotein A1 (hnRNPA1), thereby suppressing cellular ferroptosis. Beyond ferroptosis, integrated O_2_ and H_2_ gas therapy assisted by GPM microneedles accelerates diabetic wound healing through promoting fibroblast proliferation, enhancing angiogenesis, reducing oxidative stress, and modulating immune responses. In this study, a series of dry and wet assays will be performed to identify the definitive mechanisms involved. The findings will define a novel TNFAIP3/hnRNPA1 ubiquitination/ferroptosis signaling axis, offering robust validation of the proposed mechanisms. In conclusion, this study will yield a ferroptosis‐targeting microneedle material with well‐defined molecular mechanisms for diabetic wound management.

## 2 Results

### 2.1 Synthesis of PMP Nanoparticles With a Spatially Separated Structure

**FIGURE 1:**
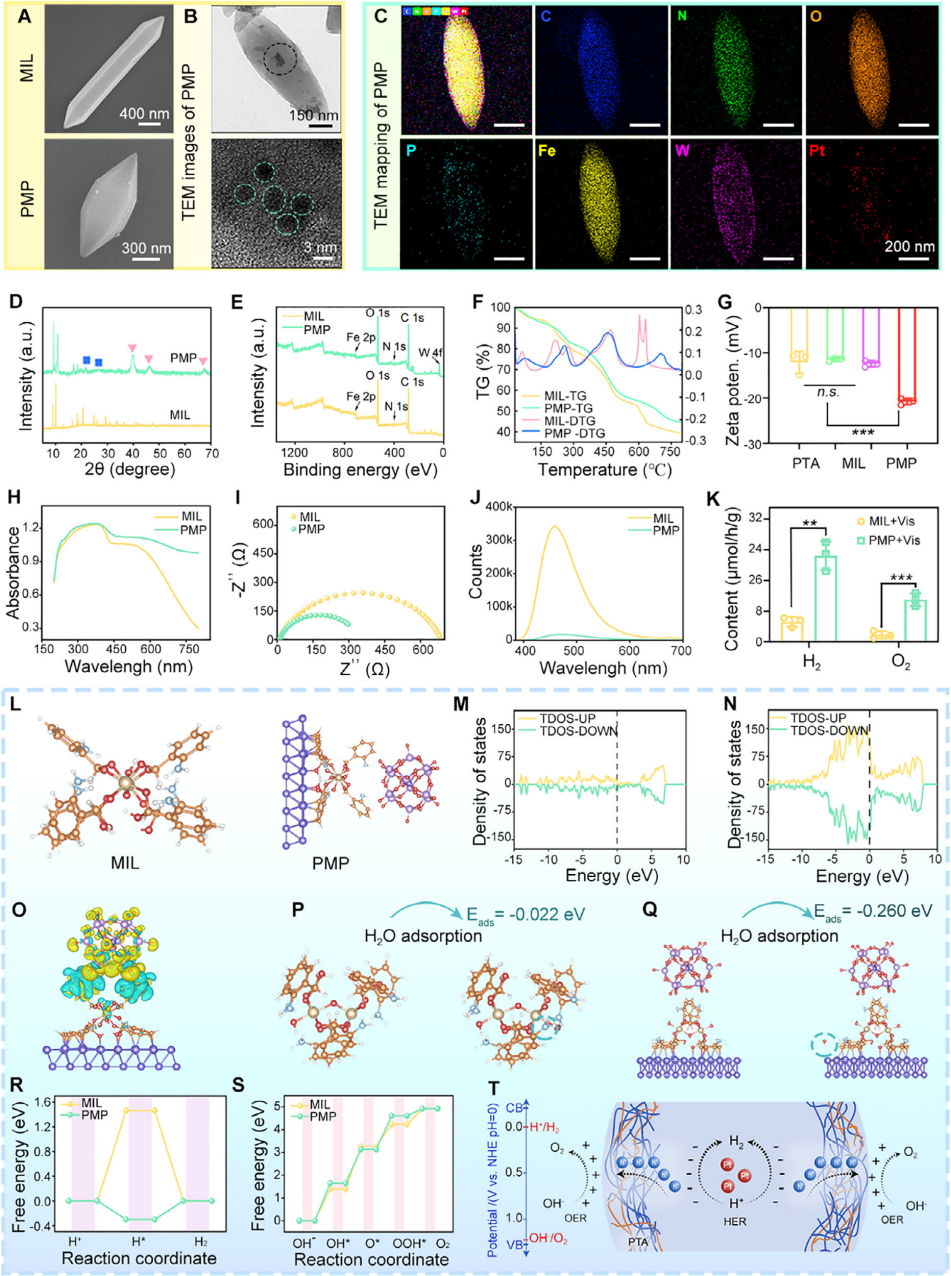
The synthesis and characterization of PMP nanoparticles. (A) SEM images of MIL and PMP. Scale bar: 400 and 300 nm; (B) TEM images of PMP. Scale bar: 150 and 3 nm; (C) Energy dispersive spectroscopy (EDS) mapping of PMP. Scale bar: 200 nm; (D) XRD patterns; (E) XPS survey spectra; (F) TG analysis; (G) Zeta potential (n = 3); (H) UV–vis absorption spectra; (I) EIS plots; (J) PL spectra; (K) H_2_ and O_2_ evolution rates under visible light irradiation (n = 3); (L) Molecular models of MIL and PMP; (M,N)  DOS plots; (O) Differential charge density of PMP; (P,Q) Values of E_ads_; (R) Gibbs free energy variation diagram for HER; (S) Gibbs free energy variation diagram for OER; (T) A diagram showing the photocatalytic mechanisms for overall water splitting. All data are presented as the mean ± SD. Statistical significance was determined using one‐way ANOVA. *n.s*. indicates no significance, ^**^p < 0.01, ^***^p < 0.001.

In this study, PMP nanoparticles featuring a spatially separated structure were designed. MIL‐101(Fe)‐NH_2_ MOFs (namely MIL MOFs) served as the host, with Pt nanoparticles encapsulated inside and PTA loaded on the exterior. The morphology of PMP nanoparticles was observed by scanning electron microscope (SEM) and transmission electron microscope (TEM). As shown in Figure 1A, both MIL and PMP exhibited a typical octahedral structure, which arises from the coordination between iron ions and the carboxyl groups in the ligands [19]. Further statistical analysis of the length distribution of nanoparticles (Figure S1) revealed that MIL had a mean length of 2.31 ± 0.39 µm, PM had a mean length of 0.83 ± 0.17 µm, and PMP had a mean length of 0.82 ± 0.18 µm. Compared to MIL, PMP exhibited smaller particle sizes and a more irregular structure. This phenomenon suggests that the incorporation of Pt nanoparticles has altered the crystallization process. As shown in Figure 1B, Pt nanoparticles, indicated by green circles, were encapsulated inside the PMP. As shown in Figure 1C, the surface of PMP nanoparticles was composed of Fe, C, N, O, Pt, P and W elements. The existence of P and W elements was attributed to PTA.

Figure 1D shows the x‐ray diffraction (XRD) patterns. Standard patterns from the Cambridge Crystallographic Data Centre (CCDC) database were used as the control (Figure S2). The peaks at 2θ of 9.2°, 10.2°, 16.8°, and 18.4° in both MIL and PMP corresponded to the (002), (101), (103), and (200) crystal planes of the MOFs, respectively [20]. The PMP nanoparticles also displayed characteristic peaks of PTA at 2θ values of 21.5° and 26.4°, which are assigned to the (220) and (222) facets, respectively [21]. Figure S3 shows the Fourier transform infrared (FT‐IR) spectrum. The peaks at 578^−^, 767^−^, 1257^−^, 1693 cm^−1^ were attributed to Fe─O stretching vibration, Fe_3_(µ_3_‐O) vibration, C─N vibration and C═O stretching vibration, respectively. The peaks at 3386^−^ and 3463 cm^−1^ were assigned to N─H stretching vibrations of amino group. PMP nanoparticles exhibited the characteristic peaks of MIL MOFs, along with those of PTA (located at 975^−^, 767^−^, and 1057 cm^−1^) [22].

Figure 1E and Figure S4 show the x‐ray photon‐electron spectroscopy (XPS) spectrum. Five element peaks were observed. Instead, no characteristic Pt peaks were detected, owning to the encapsulation of Pt within the PMP matrix and the limited probing depth of XPS (less than 10 nm) [23]. The high‐resolution spectrum of Fe 2p revealed the characteristic peaks at 713 and 726 eV, corresponding to the Fe 2p_1/2_ and Fe 2p_3/2_ peaks of PMP nanoparticles [24, 25, 26]. PMP nanoparticles exhibited three characteristic peaks of C 1s at 284.8, 285.8, and 288.8 eV, which are attributed to the C‐C bonds in the benzene ring and carboxylate structure, C─O bonds, and C═O bonds in the ligand, respectively. PMP nanoparticles also exhibited two characteristic peaks of O 1s at 532 and 532.7 eV, corresponding to the C─O─Fe and H─O─Fe bonds, respectively.

Figure 1F shows the thermogravimetric (TG) curves. The weight loss of PMP nanoparticles at 75°C and 255°C corresponded to the evaporation of free and combined water within the pores of MOFs, respectively [27]. The water desorption temperatures of PMP (75°C and 255°C) were higher than those of MIL (60°C and 211°C), indicating stronger water binding capacity. This property renders the PMP nanoparticles favorable for overall water splitting. Figure 1G and Figures S5 and S6 show the results of Zeta potential and Brunner‐Emmet‐Teller (BET) tests. PMP nanoparticles exhibited a more negative zeta potential (*p* < 0.001). PMP nanoparticles also exhibited a larger specific surface area, providing more active sites for photocatalysis.

In summary, these results have demonstrated the successful synthesis of PMP nanoparticles with a spatially separated structure.

### 2.2 PMP Nanoparticles Catalyzed Overall Water Splitting

PMP nanoparticles are anticipated to demonstrate photocatalytic potential for overall water splitting. To verify this hypothesis, a series of physicochemical characterizations were conducted.

Figure 1H shows the results of UV–vis diffuse reflectance spectroscopy (UV–vis DRS). Compared to MIL, PMP exhibited strong absorption over a broader spectral range. Such extended light harvesting enables more efficient utilization of light energy, particularly in the visible‐light range. As shown in Figure S7, the bandgap of MIL and PMP was calculated using a Tauc‐Plot method [28]. It was 1.671 eV for MIL MOFs and 1.479 eV for PMP nanoparticles, respectively. The narrowing of the bandgap reduces the energy required for electron excitation, thus promoting the transition of photogenerated electrons from the valence band to the conduction band [29]. As shown in Figure S8, the valence band (VB) of PMP nanoparticles was 1.27 eV. It was further calculated that the conduction band (CB) of PMP nanoparticles was −0.209 eV.

Figure 1I and Figure S9 show the electrochemical impedance spectroscopy (EIS), linear sweep voltammetry (LSV) curves and i‐t curves, respectively. Compared to MIL, PMP exhibited a higher photocurrent density and lower impedance, suggesting more efficient generation of photogenerated charge carriers under visible light irradiation. Moreover, the lower impedance of PMP nanoparticles minimizes the energy loss during charge carrier migration. The i‐t curves further confirmed this result: under visible light irradiation, the photocurrent density of PMP was not only significantly higher than that of MIL, but also remained almost unchanged after six consecutive light on‐off cycles, further confirming the excellent stability of PMP under visible light irradiation. Figure 1J shows the photoluminescence (PL) spectrum. The substantially reduced PL intensity of PMP nanoparticles suggested a lower electron‐hole recombination rate, allowing a larger fraction of photogenerated charge carriers to be retained for subsequent photocatalytic processes. To further probe the charge transfer dynamics, we carried out time‐resolved photoluminescence (TRPL) decay measurements (Figure S10). The decay curves were fitted using a triple‐exponential function, and the average fluorescence lifetimes were calculated accordingly. Notably, the average lifetime of PMP was found to be significantly prolonged to 1.241 ns compared with that of MIL (0.639 ns), clearly indicating that the interfacial charge transfer has been effectively improved due to the advantageous spatial structure of PMP. In conclusion, these results have demonstrated the enhanced photocatalytic potential of PMP nanoparticles.

For overall water splitting, the relevant potentials are 0 eV (vs. NHE) for hydrogen evolution reaction (HER) and 1.23 eV (vs. NHE) for oxygen evolution reaction (OER) [30]. OER is the rate‐determining step (RDS). The VB of PMP is more positive than the OER potential, indicating sufficient thermodynamic driving force for water oxidation. Meanwhile, the CB of PMP lies below the HER potential, enabling proton reduction. Thus, PMP nanoparticles possess the thermodynamic potential to catalyze both the OER and HER for overall water splitting. As shown in Figure 1K, the H_2_‐ and O_2_‐production were confirmed by gas chromatography. Under visible light irradiation, the H_2_‐ and O_2_‐producing rates of MIL MOFs were 5.21 ± 1.29^−^ and 1.84 ± 0.94 µmol·h^−1^·g^−1^, respectively. Meanwhile, those of PMP nanoparticles reached to 22.43 ± 3.78^−^ and 10.99 ± 1.64 µmol·h^−1^·g^−1^, representing a 4.3‐fold and 12.2‐fold improvement, respectively.

### 2.3 Mechanistic Insights Into Overall Water Splitting

The enhanced photocatalytic overall water splitting of PMP nanoparticles results from the rational design of a spatially separated structure. The H_2_ evolution co‐catalyst (Pt nanoparticles) and the O_2_ evolution co‐catalyst (PTA) are located inside and outside the MIL MOFs, forming a sandwich‐like heterojunction interface. This configuration narrows the bandgap of the composite PMP nanoparticles. Moreover, it establishes spatially distinct HER and OER centers. Photogenerated electrons migrate to the internal Pt nanoparticles (HER center), while holes migrate to the external PTA (OER center). In summary, the spatially separated structure not only enhances the generation and migration of electron‐hole pairs, but also suppresses their recombination. The experimental findings of this study align well with the foregoing theoretical analysis.

The proposed mechanisms were further verified using a density functional theory (DFT) calculation. As shown in Figure 1L, the structural models of MIL and PMP were established. Figure 1M,N show the density of states (DOS) plots for MIL and PMP, respectively. Both DOS profiles crossed the Fermi level, demonstrating excellent metallic conductivity that facilitates the migration of charge carriers [31]. Moreover, the larger integrated DOS area reflected more electronic states near the Fermi level, thereby facilitating electron transitions [32]. As shown in Figure 1O, electron density accumulated in PMP nanoparticles, suggesting that photogenerated charge carriers were migrated and accumulated [33].

As shown in Figure 1P,Q, the water adsorption energy (E_ads_) of PMP nanoparticles (−0.26 eV) is more negative than that of MIL MOFs (−0.022 eV). Thus, PMP enables efficient capture of water molecules, a critical step for overall water splitting [34]. Figure 1R,S and Tables S1 and S2 show the Gibbs free energy for HER and OER. The incorporation of Pt nanoparticles inside the PMP has reduced the activation energy required for HER. For the OER, the RDS in MIL MOFs is OH^*^ → O^*^, with a Gibbs free energy of 1.863 eV, whereas in PMP nanoparticles, the RDS is OH^−^ → OH^*^, with a Gibbs free energy of 1.649 eV. The lower Gibbs free energy confirms that the OER proceeds more readily in PMP nanoparticles.

Figure 1T summarizes the photocatalytic mechanisms for overall water splitting. Under visible light irradiation, PMP nanoparticles efficiently catalyze the overall water splitting to generate O_2_ and H_2_. These O_2_ and H_2_, featuring controllability and biosafety, pave the way for integrated gas therapy in biomedical applications.

### 2.4 Preparation of PMP‐Laden GP‐n Hydrogels

In this study, PMP nanoparticles are used for accelerating diabetic wound healing by integrated O_2_ and H_2_ gas therapy. Although PMP nanoparticles have demonstrated good photocatalytic activity for O_2_ and H_2_ generation, their biomedical applications remain limited. PMP nanoparticles are insoluble in water and prone to aggregation, posing significant challenges for wound administration. To address these challenges, it is necessary to modify the PMP nanoparticles by fabricating composite biomaterials.

**FIGURE 2:**
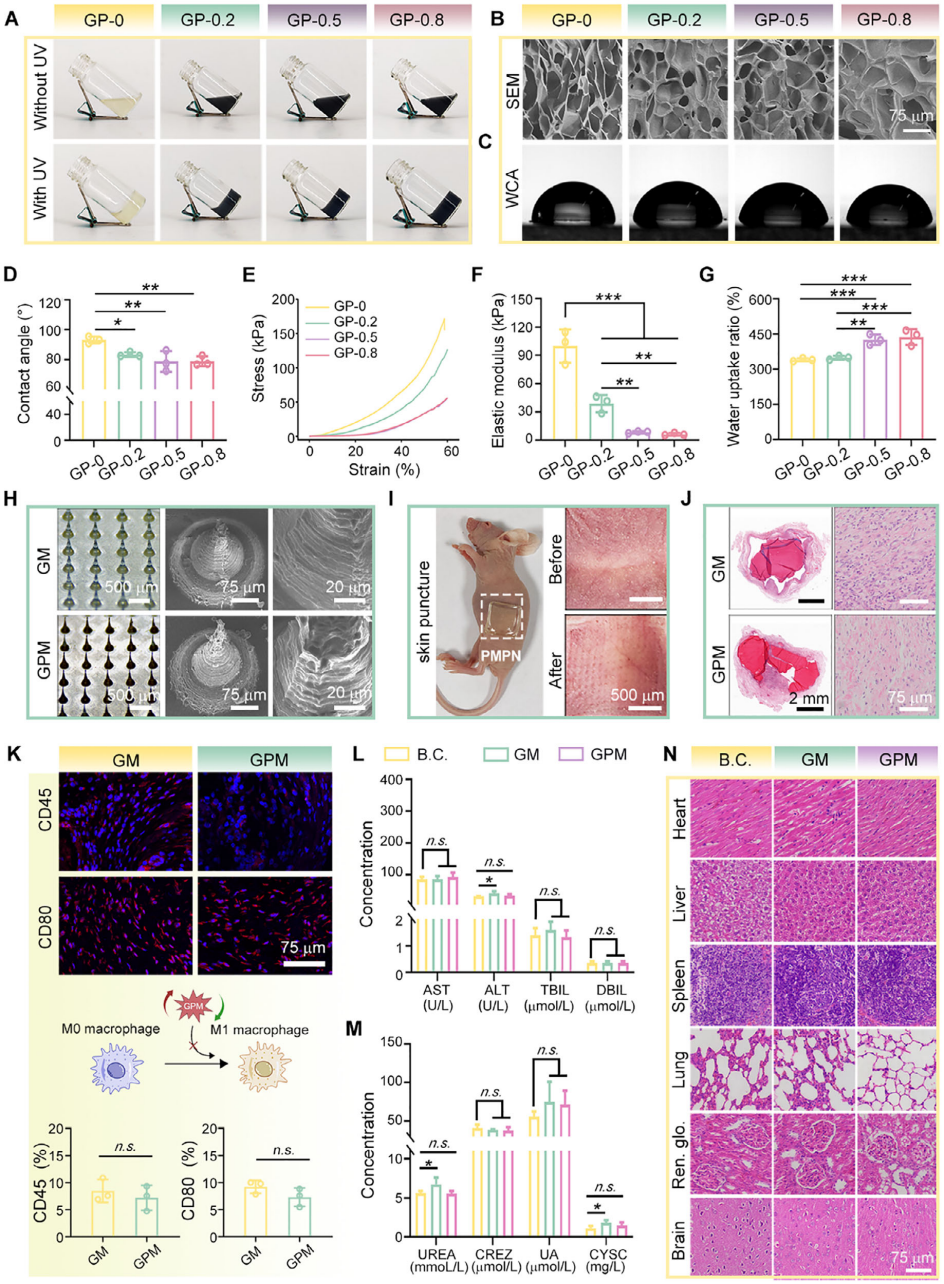
The fabrication and biocompatibility evaluations of GPM microneedles. (A) Gelation process; (B) SEM images of freeze‐dried hydrogels. Scale bar: 75 µm; (C,D) Water contact angle (n = 3); (E,F) Compression test (n = 3); (G) Equilibrium swelling ratio (n = 3); (H) Morphology of GM and GPM microneedles. Scale bar was marked; (I) Skin puncturing test of GPM microneedles. Scale bar: 500 µm; (J) H&E staining images of the microneedles and the capsules. Scale bar: 2 mm or 75 µm; (K) Immunofluorescence (IF) staining images (CD45 and CD80) and the quantitative results of capsule tissues (n = 3). Scale bar: 75 µm; (L,M) Blood biochemical tests (n = 3); (N) H&E staining images of the organs. Scale bar: 75 µm. All data are presented as the mean ± SD. Statistical significance was determined using one‐way ANOVA. *n.s*. indicates no significance, ^*^p < 0.05, ^**^p < 0.01, ^***^p < 0.001.

As shown in Figure 2A, PMP nanoparticles were modified with GelMA hydrogels. Four kinds of PMP‐laden GelMA hydrogels (namely GP‐n, n corresponding to the content of PMP) were prepared using an ultraviolet (UV)‐crosslinking strategy. GelMA was chemically crosslinked to form a molecular skeleton, within which PMP nanoparticles were physically encapsulated. The freeze‐dried hydrogels exhibited a porous structure, which is beneficial for absorbing wound exudates (Figure 2B). As the content of PMP nanoparticles increased, the hydrophilicity of GP‐n hydrogels got significantly improved (Figure 2C,D). This improvement is attributed to the reduced crosslinking density caused by PMP interference with photoinitiator radical regeneration [35].

Figure 2E,F show the results of compression test. The stress of hydrogels increased within the strain range of 0%–‐60 %, demonstrating favorable elastic deformation. The elastic modulus was 99.63 ± 17.62 kPa for GP‐0, 38.64 ± 9.1 kPa for GP‐0.2, 8.47 ± 1.02 kPa for GP‐0.5, and 6.29 ± 1.37 kPa for GP‐0.8, respectively. Compared to GP‐0 group, the incorporation of PMP nanoparticles significantly reduced the elastic modulus (*p* < 0.001). PMP nanoparticles interfered with the free radicals regenerated by the photo‐initiator, thereby reducing the crosslinking degree and mechanical strength of composite hydrogels.

Figure 2G shows the results of swelling test. As the content of PMP nanoparticles increased, the equilibrium swelling ratio of GP‐n hydrogels exhibited a gradual rise. PMP nanoparticles altered the gelation state of GP‐n hydrogels, leading to a looser structure and increased porosity. Consequently, the swelling performance of GP‐n hydrogels was improved. When used as wound dressings, GP‐n hydrogels rapidly and efficiently absorb wound exudates, thereby maintaining a clean, moist microenvironment to support tissue regeneration.

In summary, four kinds of PMP‐laden GP‐n hydrogels were successfully prepared, featuring decreased mechanical strength but improved hydrophilicity and swelling ability.

### 2.5 Fabrication of Biocompatible GPM Microneedles

To improve the delivery efficiency of PMP nanoparticles, GP‐n hydrogels were further processed into bilayer GPM microneedles (Figure 2H). The base layer and tip layer of GPM microneedles were composed of GP‐0 hydrogels and GP‐0.5 hydrogels, respectively. As the control, both the base and tip layers of GM microneedles were composed of GP‐0 hydrogels. For the base layer, the hydrogels themselves not only protect wounds from secondary injury but also provide essential cues to promote skin regeneration. To comprehensively evaluate the microneedle penetration capability, we first performed compressive mechanical testing on the microneedles (Figure S11). Both types of microneedles exhibited satisfactory mechanical strength at their tips. The maximum load‐bearing capacity of a single GM microneedle tip was 0.169 N, while that of a single GPM microneedle tip was 0.122 N. Both values exceeded the required penetration threshold of 0.098 N per needle tip [36]. Subsequently, BALB/c nude mice skin was used to evaluate the microneedle penetration performance. As shown in Figure 2I and Figure S12, the sharp tips of GPM microneedles punctured the skin tissues of BALB/c nude mice. In the tip layer, PMP nanoparticles are delivered to the wound site in an array pattern. To evaluate the drug delivery efficiency of the GPM microneedle tip layer, we used rhodamine B (Rh.B) as a fluorescent tracer to replace PMP nanoparticles and fabricated bilayer microneedles (GRM) with Rh.B‐loaded tips, and then simulated the drug release process in vitro [37]. First, a calibration curve of Rh.B absorbance was constructed by measuring the absorbance of Rh.B standard solutions at various concentrations at 550 nm (Figure S13A). Subsequently, the GRM were immersed in release medium, and the absorbance of the medium was measured at different time points to calculate the cumulative amount of drug released at each time point (Figure S13B). The results showed that GRM exhibited a rapid release within the first 2 h, followed by a gradually decelerated release and reached a plateau at 12 h. This release profile indicates that when the microneedles are applied to skin wounds, the active components can be rapidly delivered to the wound site, enabling efficient early‐stage drug administration.

The biocompatibility of GPM microneedles was evaluated using a subcutaneous implantation model of Sprague Dawley (SD) rats. Blank control (B.C.) group was sham‐operated. As shown in Figure 2J, Subcutaneous tissue rapidly responded to the embedded foreign objects by forming a fibrous capsule to envelop them. Compared to GM group, less inflammatory cells were found in GPM group, suggesting good tissue‐compatibility. As shown in Figure 2K and Figure S14, all experimental groups exhibited low positive rate of inflammatory‐related markers (CD45, CD80 and CD206). No significant difference was observed between GM and GPM groups (*p* > 0.05). These results further confirmed the tissue‐compatibility of the microneedle materials.

Both GM and GPM microneedles undergo partial biodegradation through a series of endogenous enzymatic approaches. The degradation products are then transported via the bloodstream to various tissues and organs, where they may affect physiological functions. Herein, the organ‐ and hemo‐compatibility were comprehensively investigated. As shown in Figure 2L,M and Figure S15, fresh blood was collected for detecting a series of biochemical indexes. For each index, no significant difference was observed (*p* > 0.05). As shown in Figure 2N, the organs of treated animals were resected and analyzed by H&E staining assay. Compared to B.C. group, GM and GPM groups exhibited no obvious pathological changes. These results indicated that the degradation products had no adverse effects.

In conclusion, the bilayer GPM microneedles exhibited high delivery efficiency of PMP nanoparticles and favorable biocompatibility, positioning them as a highly promising platform for integrated O_2_ and H_2_ gas therapy. However, their therapeutic effects and underlying mechanisms in accelerating diabetic wound healing remain to be further explored.

### 2.6 GPM Microneedles Accelerated Diabetic Wound Healing in Vivo

**FIGURE 3:**
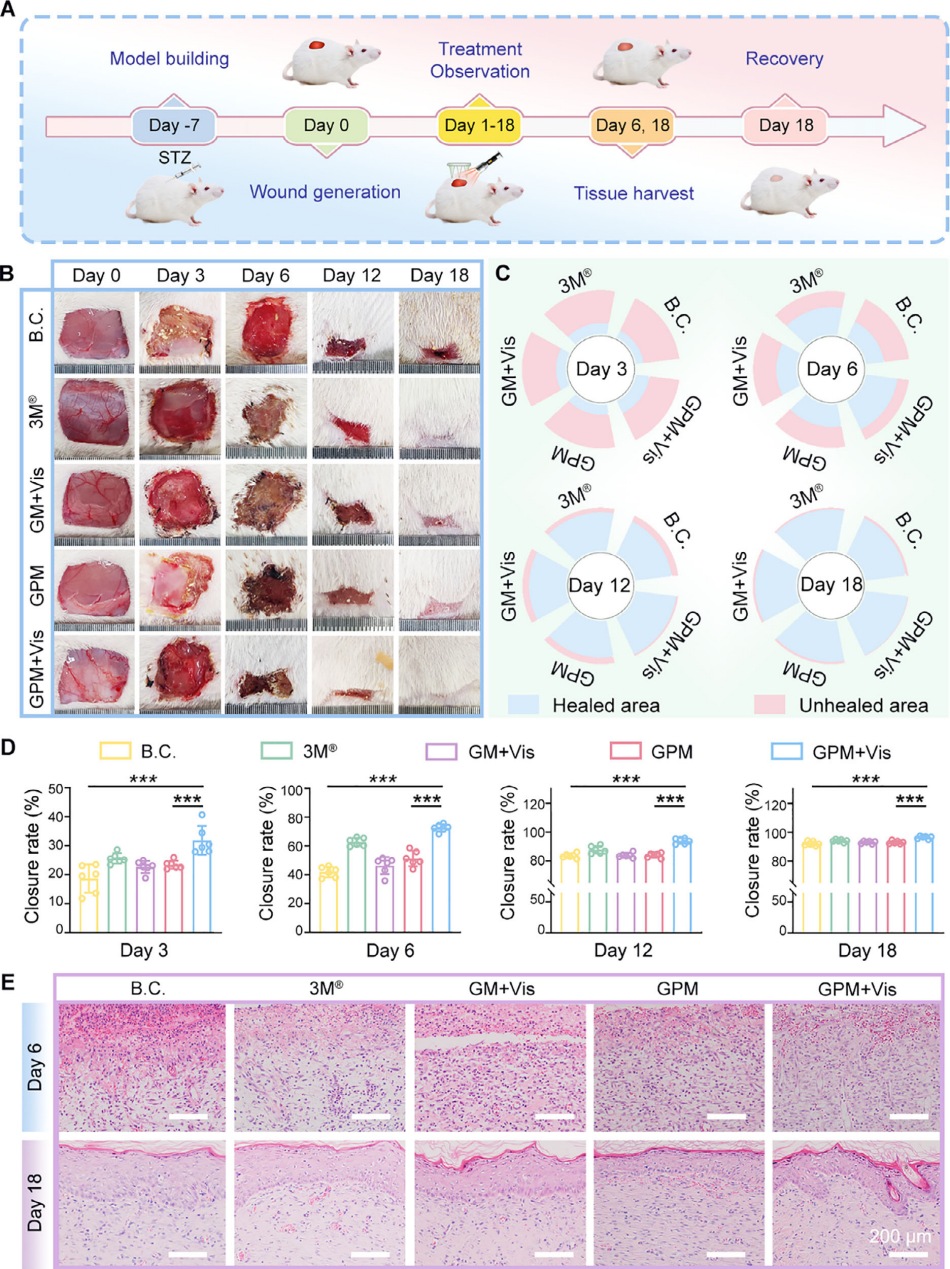
GPM microneedles accelerated diabetic wound healing in a rat model. (A) A diagram showing the experimental plan; (B) Representative images of wound sites; (C) Traces of wound area; (D) Quantitative results of wound closure rate at each time point (n = 6); (E) H&E staining images of neo‐skin tissue. Scale bar: 200 µm. All data are presented as the mean ± SD. Statistical significance was determined using one‐way ANOVA. ^***^p < 0.001.

As shown in Figure S16A,B, a diabetic SD rat model was established by high‐sugar, high‐fat diet and intraperitoneal streptozotocin (STZ) administration [38]. Figure 3A shows the overall protocols of wound healing assay. A full thickness skin defect was created on each animal. The wounds were treated with GPM microneedles with or without visible light irradiation (GPM group and GPM+Vis Group). The positive control was treated with commercial wound dressings (3M group), the negative control was treated with GM group with visible light irradiation (GM+Vis group), the blank control was not treated (B.C. group).

Figure 3B,C show the photographs of wound sites and their wound healing rate at varied time points. The wounds completed wound closure and skin regeneration within 18 days. The quantitative results of wound closure rate are shown in Figure 3D. At each time point, GPM+Vis group healed fastest among the five groups. For example, the closure rate at Day 6 was 46.4% ± 5.9% for B.C. group, 62.7% ± 2.8% for 3M group, 41.9% ± 3.6% for GM+Vis group, 51.3% ± 5.3% for GPM group, and 72.5% ± 2.5% for GPM+Vis group, respectively. Significant difference was observed between 3M group and GPM+Vis group (*p* < 0.001). This phenomenon suggested that the pro‐regeneration efficiency of GPM+Vis treatment is better than that of a commercial wound dressing.

Neo‐skin tissues at Day 6 and Day 18 were evaluated by a series of histological staining assays. Figure 3E shows the H&E staining images. At Day 6, granulation tissue filled the wounds. A large number of capillaries and red blood cells were found in the granulation tissue, providing necessary nutrition and O_2_ for tissue regeneration. Compared to other groups, less inflammatory cells and inflammatory exudates were observed in GPM+Vis group. This phenomenon could be attributed to the good biocompatibility of GPM microneedles themselves, as well as the anti‐inflammatory effect of integrated O_2_ and H_2_ gas therapy. At Day 18, all groups exhibited complete keratinizing epithelium, indicating that wound closure process was finished. Compared to other groups, GPM+Vis group exhibited more matured dermal, epithelium and hair follicles.

### 2.7 GPM Microneedles Demonstrated Multiple Pro‐Regenerative Activities

**FIGURE 4:**
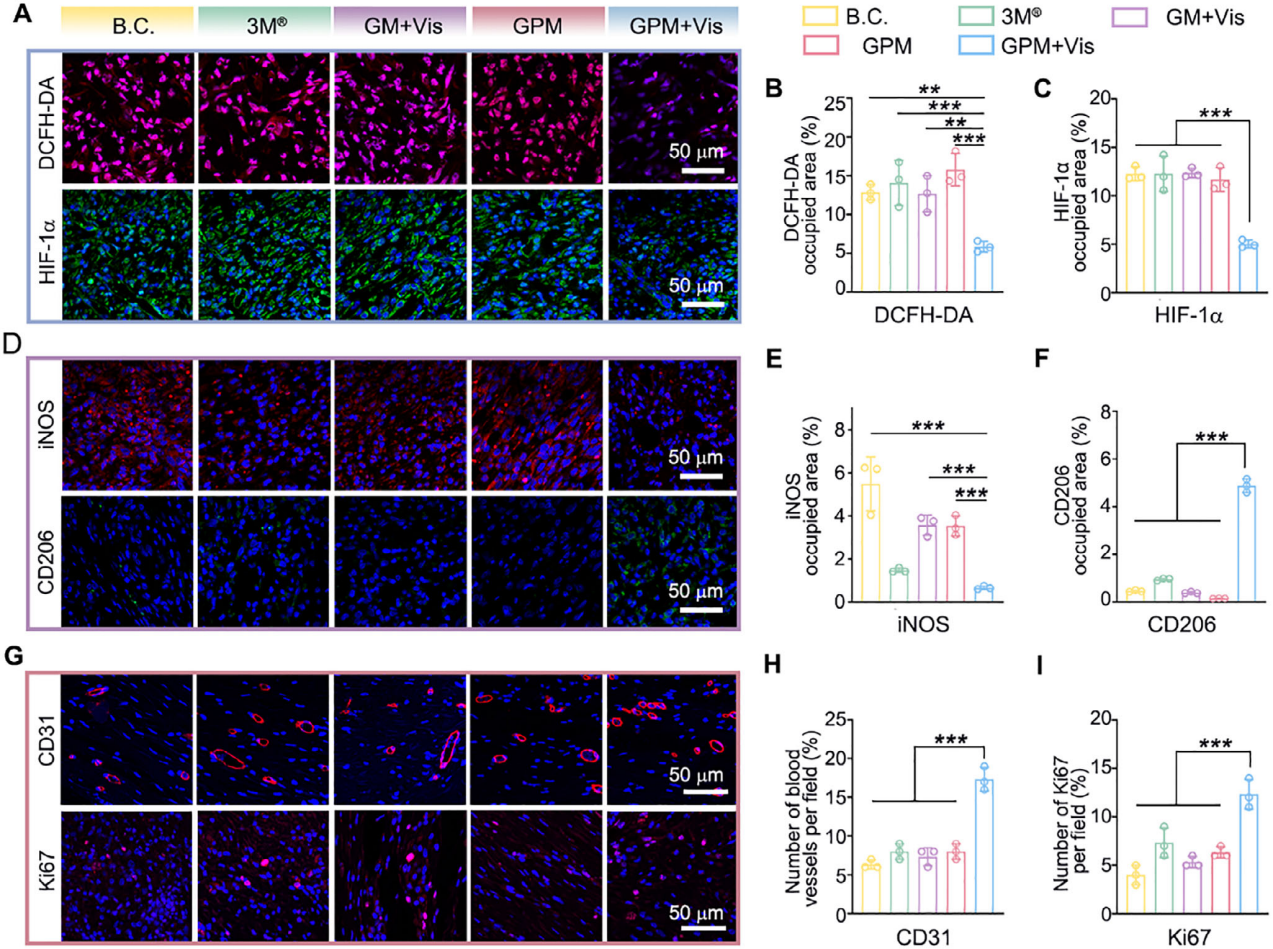
Histological evaluations revealing the pro‐regenerative activities of GPM microneedles. (A) IF images of DCFH‐DA and HIF‐1α at Day 6. Scale bar: 50 µm; (B,C) Quantitative results of DCFH‐DA and HIF‐1α (n = 3); (D) IF images of iNOS and CD206 at Day 6. Scale bar: 50 or 100 µm; (E,F) Quantitative results of iNOS and CD206 (n = 3); (G) IF images of CD31 and Ki67 at Day 18. Scale bar: 50 µm; (H,I) Quantitative results of CD31 and Ki‐67 (n = 3). All data are presented as the mean ± SD. Statistical significance was determined using one‐way ANOVA. ^**^p < 0.01, ^***^p < 0.001.

A series of histological staining assays were performed to explore the pro‐regenerative activities of GPM microneedles. As shown in Figure 4A,C, the GPM+Vis treatment significantly reduced the expression levels of DCFH‐DA (a marker of the oxidative stress) and HIF‐1α (a marker of hypoxia). This phenomenon is attributed to the critical roles of integrated O_2_ and H_2_ gas therapy. As a signaling molecule, H_2_ gas activates a range of antioxidant enzymes such as catalase (CAT) and superoxide dismutase (SOD), thereby reducing ROS levels and oxidative stress injury [9]. O_2_ gas down‐regulates the HIF‐1α signaling pathway for ameliorating the hypoxia in diabetic wounds [39].

In diabetes, hyperglycemia disrupts the balance between pro‐inflammatory M1 macrophages and pro‐regenerative M2 macrophages [40]. The induction of M2 macrophage polarization is generally considered a promising approach for promoting tissue regeneration. Macrophage polarization was evaluated by immunofluorescence staining of M1 and M2 markers [41, 42, 43]. Herein, we found that GPM+Vis treatment inhibited the M1 macrophage polarization (marked by iNOS) and promoted the M2 macrophage polarization (marked by CD206) (Figure 4D–F). As shown in Figure 4G–I and Figure S17A–C, GPM+Vis treatment also promoted cell proliferation (marked by Ki67), angiogenesis (marked by CD31), and tissue remodeling (marked by Col‐I and Col‐III).

Under visible light irradiation, GPM microneedles demonstrated multiple pro‐regenerative activities: anti‐oxidation, anti‐hypoxia, induction of M2 macrophage polarization, promotion of cell proliferation, angiogenesis, and tissue remodeling. These activities were attributed to the intrinsic properties of the GelMA hydrogels, as well as the integrated O_2_ and H_2_ gas therapy. Previous works have widely reported the regeneration‐associated properties of GelMA hydrogels [44]. However, integrated O_2_ and H_2_ gas therapy remains an emerging concept. Little knowledge has been obtained regarding their synergistic mechanisms in therapeutic applications.

### 2.8 Integrated O_2_ and H_2_ Gas Therapy Inhibited TNFAIP3‐Mediated Ferroptosis

**FIGURE 5:**
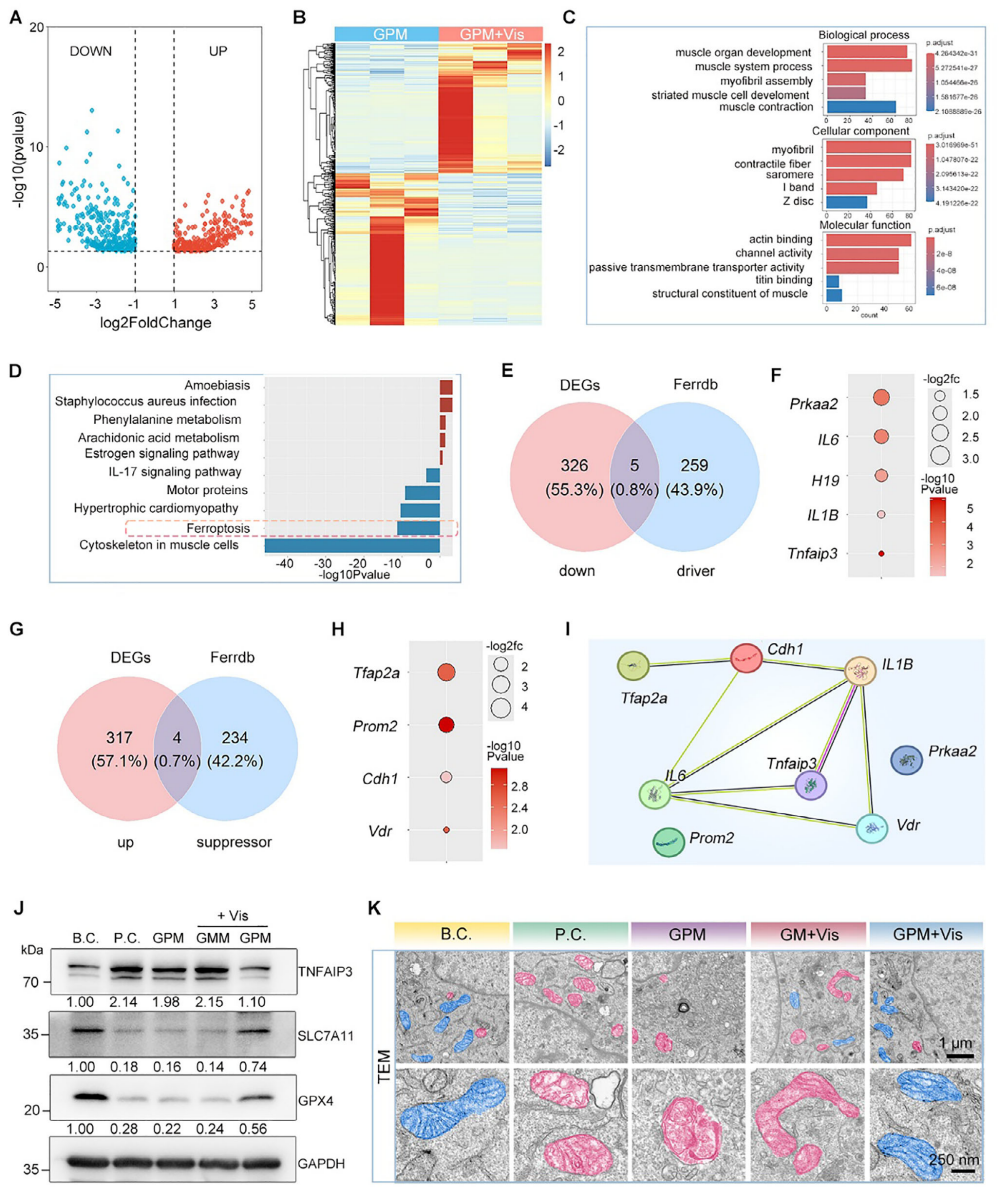
Screening out the molecular target of integrated O_2_ and H_2_ gas therapy. (A) Volcano plot of DEGs: GPM group vs. GPM+Vis group (n = 3); (B) Heatmap of DEGs (n = 3); (C) GO analysis; (D) KEGG analysis; (E,F) Bioinformatic integration of down‐regulated DEGs with the FerrDb database; (G,H) Bioinformatic integration of up‐regulated DEGs with the FerrDb database; (I) PPI network analysis; (J) Western blot for GPX4, SLC7A11 and TNFAIP3; (K) TEM images showing the ferroptosis‐associated mitochondrial morphology. Scale bar: 1 µm and 250 nm.

An RNA‐seq was performed to explore the pro‐regenerative mechanisms of integrated O_2_ and H_2_ gas therapy at the molecular levels. As shown in Figure 5A,B, two groups including GPM group and GPM+Vis group were set to eliminate the influence of GelMA hydrogels (n = 3). Compared to GPM group, GPM+Vis group exhibited 321 up‐regulated differentially expressed genes (DEGs) and 331 down‐regulated DEGs. These DEGs were modulated by integrated O_2_ and H_2_ gas therapy rather than GelMA hydrogels themselves. Gene Ontology (GO) and Kyoto Encyclopedia of Genes and Genomes (KEGG) analysis were further performed (Figure 5C,D). These DEGs were involved with a series of biological processes, cellular components, and molecular functions, such as muscle organ development, muscle system process, muscle contraction, actin binding, channel activity, passive transmembrane transporter activity. They were further enriched to a series of signal pathways, including ferroptosis.

Ferroptosis is an iron‐dependent, non‐apoptotic form of regulated cell death (RCD) [4]. Excessive activation of ferroptosis in diabetic patients significantly impedes wound healing, representing a key pathological mechanism [45]. In recent years, the biological roles of ferroptosis in regeneration medicine have attracted much attention. Fu et al. found that secretory autophagosomes inhibited cell ferroptosis by decreasing generation and increasing discharge of ferrous ion, thus accelerating diabetic wound healing [46]. Li et al. also reported that hesperetin promoted diabetic wound healing by inhibiting ferroptosis through the activation of SIRT3 [47]. These studies have identified ferroptosis as one of the promising therapeutic targets for diabetic wounds. However, a mechanistic link to integrated O_2_ and H_2_ gas therapy has yet to be established.

As shown in Figure 5E–H, integrated analysis of our RNA sequencing with a public ferroptosis database (namely FerrDb) identified 5 concordantly down‐regulated and 5 up‐regulated DEGs [48]. Prkaa2, IL6, IL1B, H19 and Tnfaip3 were the driver of ferroptosis, while Tfap2a, Prom2, Cdh1, and Vdr were the suppressor. As shown in Figure 5I, protein‐protein interaction (PPI) analysis was performed using the String database, and identified Tnfaip3 as a hub gene in the PPI network. Tnfaip3 gene encodes TNFAIP3 (tumor necrosis factor alpha‐induced protein 3), which functions in modulating immune and inflammatory responses, glutathione (GSH) depletion and ferroptosis inhibition [49]. Herein, it is assumed that TNFAIP3 is one of the protein targets of integrated O_2_ and H_2_ gas therapy to inhibit cellular ferroptosis.

As shown in Figure 5J and Figure S18, the protein expression of ferroptosis markers (GPX4 and SLC7A11) was significantly upregulated in a cell‐based model of ferroptosis (GPM+Vis group *v.s*. positive control (P.C.) group). The state of ferroptosis was rescued by GPM+Vis treatment. The expression of TNFAIP3 protein was also down‐regulated by GPM+Vis treatment, which is consistent with our hypothesis. Cellular ferroptosis is typically characterized by mitochondrial membrane condensation, volume shrinkage, outer membrane rupture, and reduction or disappearance of mitochondrial cristae [50]. As shown in Figure 5K, mitochondrial morphology was rescued by GPM+Vis treatment, further confirming its ferroptosis inhibition effect. The intracellular levels of malondialdehyde (MDA) and glutathione (GSH), which reflect lipid peroxidation‐induced oxidative stress, were used to evaluate the ferroptosis status of the cells [51]. As presented in Figure S19, cells subjected to high‐glucose culture and cobalt chloride‐induced hypoxic (P.C.) conditions showed significantly elevated MDA content and reduced GSH levels relative to the B.C., confirming the occurrence of ferroptosis. In contrast, treatment with GPM under visible light (GPM+Vis) reversed these changes, with a notable decline in MDA and an increase in GSH, thereby demonstrating that the ferroptosis state was effectively ameliorated through the GPM‐mediated gas therapy.

In conclusion, the inhibition of cellular ferroptosis by integrated O_2_ and H_2_ gas therapy is highly associated with TNFAIP3 downregulation, implying a potential functional interaction. Nevertheless, the mechanism by which TNFAIP3 modulates the ferroptosis pathway requires further investigation.

### 2.9 TNFAIP3 Regulated Ferroptosis by the Ubiquitination of hnRNPA1

**FIGURE 6:**
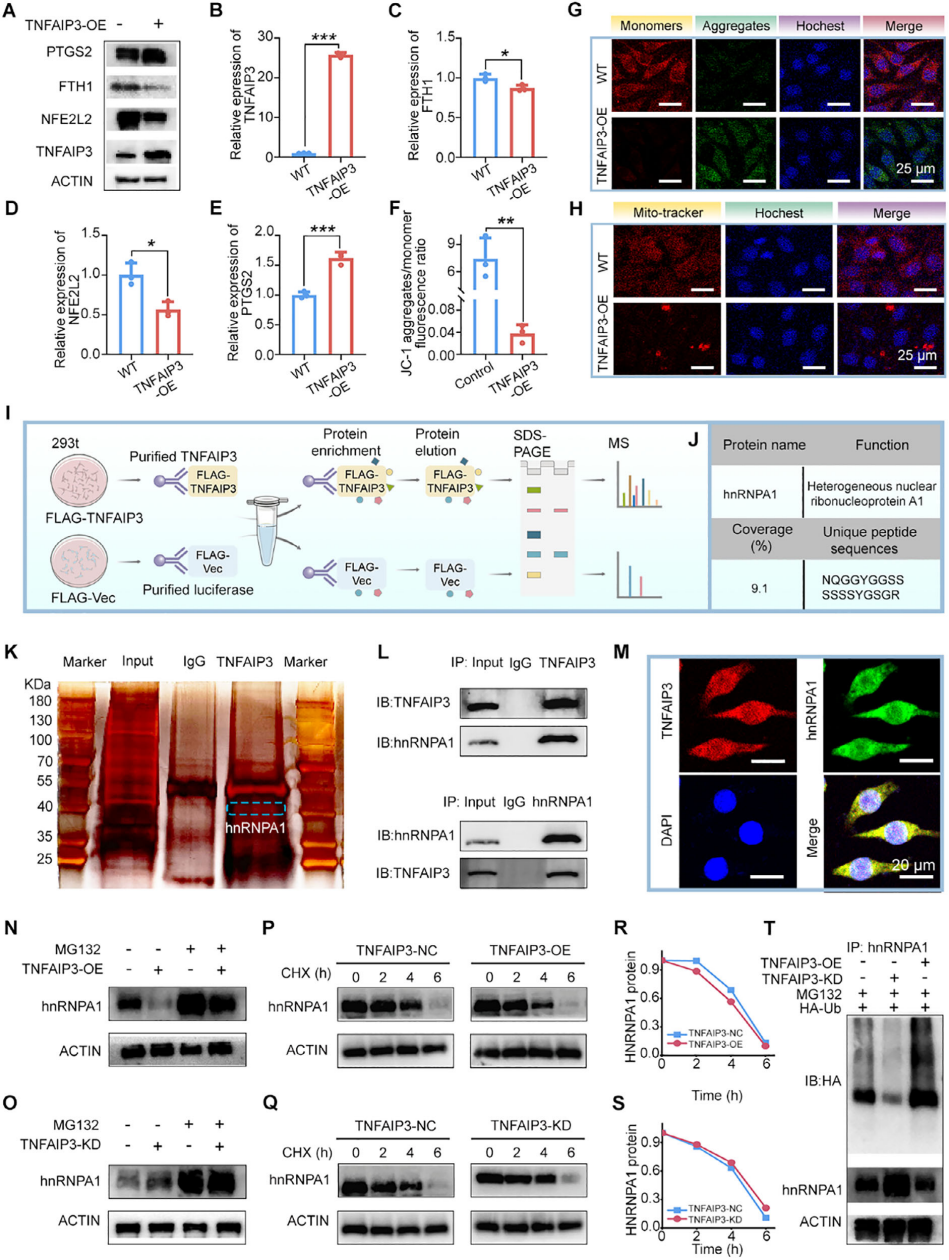
Verification of the mechanism of TNFAIP3/hnRNPA1 ubiquitination /ferroptosis axis. (A–E) WB and qPCR analysis of TNFAIP3, PTGS2, FTH1, and NFE2L2 (n = 3); (F,G) Mitochondrial membrane potential assay with JC‐1 (n = 3). Scale bar: 25 µm; (H) MitoTracker red staining assay. Scale bar: 25 µm; (I) A diagram showing the protocols of co‐IP assay followed by MS analysis; (J) hnRNPA1 was screened out; (K) SDS‐PAGE assay; (L) Direct binding between hnRNPA1 and TNFAIP3; (M) Co‐localization assay. Scale bar: 20 µm; (N,O) Western blot of hnRNPA1 expression with or without MG132 treatment in TNFAIP3‐OE and TNFAIP3‐KD cells; (P–S) Western blot and quantification of hnRNPA1 in CHX‐treated TNFAIP3‐OE and TNFAIP3‐KD cells (n = 3); (T) Western blot of the ubiquitination of hnRNPA1. All data are presented as the mean ± SD. Statistical significance was determined using one‐way ANOVA. ^*^p < 0.05, ^**^p < 0.01, ^***^p < 0.001.

To explore the downstream mechanism of TNFAIP3, the fibroblasts (L929 cells) were transfected with TNFAIP3‐overexpressed plasmid (TNFAIP3‐OE group). L929 cells represent to be a standard cell line for wound healing evaluations. However, compared to primary dermal fibroblasts or diabetic‐source cells, L929 cells have limitations in fully representing primary fibroblasts in diabetic wounds. The wild type (WT) group was transfected with empty vector and used as the control. As shown in Figure 6A, the protein expression of TNFAIP3 was significantly higher in the TNFAIP3‐OE group than in the WT group, suggesting that cell model was successfully established. Meanwhile, TNFAIP3‐OE group showed upregulation of prostaglandin‐endoperoxide synthase 2 (PTGS2) protein and downregulation of both ferritin heavy chain 1 (FTH1) protein and NFE2‐like basic leucine zipper transcription factor 2 (NFE2L2) protein. The mRNA expression exhibited similar trend (Figure 6B–E). These results confirmed that the overexpression of TNFAIP3 promotes cellular ferroptosis.

Mitochondria play a vital role in ferroptosis, primarily by regulating intracellular iron metabolism and ROS levels to influence cell fate [50]. In this study, the mitochondrial membrane potential was detected using a JC‐1 staining kit (Figure 6F,G). The monomers were dyed green, and J‐aggregates were dyed red. Compared with WT group, TNFAIP3‐OE group exhibited reduced red fluorescence intensity, indicating a decrease in mitochondrial membrane potential. As shown in Figure 6H, the biodistribution of mitochondrion was also visualized using a MitoTracker red staining assay. For WT group, mitochondria were evenly distributed in the cytoplasm. For TNFAIP3‐OE group, mitochondria shrank and aggregated into dots. These results further confirmed that the overexpression of TNFAIP3 induced mitochondrial damage and promoted cellular ferroptosis.

As shown in Figure 6I, a combined technique of co‐immunoprecipitation (co‐IP) and mass spectrometry (MS) was applied for screening the interacting proteins of TNFAIP3. The results demonstrated heterogeneous nuclear ribonucleoprotein A1 (hnRNPA1) as a candidate with a coverage of 9.1% (Figure 6J,K). hnRNPA1 is recognized for its critical roles in regulating mRNA synthesis, stability, translation, and packaging into extracellular vesicles [52]. Additionally, hnRNPA1 is involved in regulating the cellular process of ferroptosis, where it exerts an inhibitory effect [53]. As shown in Figure 6L, the combination of hnRNPA1 and TNFAIP3 was confirmed by co‐IP assay. Both the hnRNPA1 and TNFAIP3 were localized in cytoplasm (Figure 6M). Their biodistribution patterns were consistent. These results demonstrated that hnRNPA1 interacts with TNFAIP3 in the cytoplasm.

As shown in Figure 6N,O, the overexpression of TNFAIP3 downregulated the expression of hnRNPA1, while the knockdown of TNFAIP3 (TNFAIP3‐KD group) upregulated the expression of hnRNPA1. Treatment with the proteasome inhibitor (MG132) successfully reversed the effects of TNFAIP3. As shown in Figure 6P–S, TNFAIP3‐KD upregulated the expression of hnRNPA1 and prolonged its half‐life, while TNFAIP3‐OE downregulated the expression of hnRNPA1 and shortened its half‐life. For eukaryotic cells, the protein degradation is mediated by the ubiquitin‐proteasome system or autophagy [54]. To investigate this, the TNFAIP3‐OE and TNFAIP3‐KD fibroblasts were transfected with hemagglutinin‐tagged ubiquitin plasmid. The results in Figure 6T show that the ubiquitination of hnRNPA1 was reduced in TNFAIP3‐KD fibroblasts and increased in TNFAIP3‐OE fibroblasts. These results demonstrated that TNFAIP3 promotes the ubiquitination degradation of hnRNPA1.

In conclusion, integrated O_2_ and H_2_ gas therapy promotes diabetic wound healing via the TNFAIP3/hnRNPA1 ubiquitination/ferroptosis axis. Specifically, downregulation of TNFAIP3 stabilizes hnRNPA1 through reduced ubiquitination, thereby suppressing ferroptosis and accelerating wound healing. This study first reveals the molecular mechanism of integrated O_2_ and H_2_ gas therapy, providing a critical theoretical foundation for translational applications.

## 3 Discussion

Gas therapy is one of the promising approaches for accelerating wound healing in diabetes. In recent years, significant progresses have been made in both O_2_ gas therapy and H_2_ gas therapy, particularly in biomaterial development and mechanistic investigation, highlighting their considerable potential for clinical translation [55]. However, O_2_ and H_2_ gas therapies have been pursued in isolation, without meaningful convergence. Whether and how their synergy might be realized stands as a defining challenge for the field. To address this challenge, this study not only proposed a concept of integrated O_2_ and H_2_ gas therapy, but also engineered a bilayer GPM microneedle platform capable of photocatalytic O_2_ and H_2_ co‐generation and co‐delivery. Briefly, PMP nanoparticles were synthesized using a hydrothermal reaction, and then immobilized onto the GPM microneedles. GPM microneedles demonstrated desirable biocompatibility, transdermal nanomaterial delivery capability, enhanced photocatalytic efficiency, and complex pro‐regenerative activities. This study further elucidated the pro‐regenerative mechanism of integrated O_2_ and H_2_ gas therapy, which was identified as the TNFAIP3/hnRNPA1 ubiquitination/ferroptosis signaling axis. Notable advancements have been achieved as follows:

The rational design of a spatially separated structure boosts the efficiency of O_2_ and H_2_ co‐evolution. The generation of O_2_ and H_2_ gases through photocatalytic overall water splitting is limited by the reaction kinetics involved [56]. In this study, a spatially separated structure was designed for the PMP nanoparticles. Pt nanoparticles were embedded into the core of PMP as the HER site, while PTA was coated onto the shell of PMP as the OER site. This structure strengthened the interface effect and facilitated charge transfer, thus inhibiting the electron‐hole recombination. PMP nanoparticles exhibited superior photocatalytic potential over the pristine MIL MOFs, including a narrower bandgap, higher conductivity, and greater carrier density. Meanwhile, PMP nanoparticles achieved a 20% higher gas generation rate than competitive products such as HTON‐Pd@Gel [57]. DFT calculations further elucidated the mechanism for the enhanced photocatalytic performances, including improved water adsorption energy, and reduced Gibbs energy barriers for both HER and OER.

The fabrication of bilayer microneedles enables the transdermal delivery of PMP nanoparticles. The biomedical applications of PMP nanoparticles are constrained by several common challenges shared by many nanotherapeutic systems, including inefficient delivery efficiency and poor tissue dispersal. To solve this problem, this study fabricated a bilayer‐structured GPM microneedle. PMP nanoparticles were immobilized into the tips of GPM microneedles to enhance their transdermal delivery efficiency, reduce their dosage, and minimize potential side effects. The microneedle matrix was composed of GelMA hydrogel, which is not only biocompatible and biodegradable but also capable of accelerating wound healing by maintaining a moist environment, preventing secondary injury, and providing essential biochemical and biophysical cues to guide tissue regeneration. The pro‐regeneration effects of GPM microneedles were verified using a full‐thickness skin defect model of diabetic SD rats. The results demonstrated that the integration of PMP nanoparticles with GelMA hydrogel yielded synergistic and complementary benefits.

The TNFAIP3/hnRNPA1 ubiquitination/ferroptosis signaling axis contributes to the pro‐regenerative mechanism of integrated O_2_ and H_2_ gas therapy. Currently, integrated O_2_ and H_2_ gas therapy represents an emerging concept in the field of regeneration medicine, including diabetic wound healing. Little knowledge has been obtained regarding its synergistic mechanisms. In this study, a high‐throughput, multi‐omics approach was used to explore this question. The results showed that ferroptosis negatively impacts diabetic wound healing. Integrated O_2_ and H_2_ gas therapy suppresses cellular ferroptosis via downregulation of TNFAIP3 mRNA expression. The consequent decrease in TNFAIP3 levels inhibits the ubiquitination‐dependent degradation of hnRNPA1, thus maintaining the anti‐ferroptosis function of hnRNPA1. Collectively, these results established the molecular mechanism of integrated O_2_ and H_2_ gas therapy as a novel TNFAIP3/hnRNPA1 ubiquitination/ferroptosis signaling axis, providing theoretical insights into biomaterial‐wound healing interactions.

In conclusion, this study introduced a bioactive GPM microneedle material and a transformative concept of integrated O_2_ and H_2_ gas therapy. As a proof‐of‐concept, we have demonstrated their molecular mechanism and application potential not only in diabetic wound healing but also in other fields of regeneration medicine. Further studies are warranted regarding their long‐term biosafety and efficacy.

## 4 Materials and Methods

### 4.1 Materials

FeCl_3_·6H_2_O, 2‐aminoterephthalic acid, H_2_PtCl_6_·6H_2_O, methanol, and ethylene glycol were obtained from Aladdin Biochemical Technology Co., Ltd. (Shanghai, China). Polyvinylpyrrolidone (PVP, MW = 55000), gelatin, and methacrylic anhydride (MA), paraformaldehyde (PFA), PTA (H_3_PW_12_O_40_), sodium sulfate (Na_2_SO_4_), acetone, and isopropanol were purchased from Sinopharm Chemical Reagent Co., Ltd. (Shanghai, China). 2‐Hydroxy‐4′‐(2‐hydroxyethoxy)‐2‐methylpropiophenone (2959) was obtained from Macklin Chemical Regent Co., Ltd. (Shanghai, China). The microneedle mold was kindly provided by Zhongding Yuxuan New Material Technology Co., Ltd. (Hefei, China). L929 cells were kindly provided by the Medical Research Center, Zhongnan Hospital of Wuhan University. Cell culture medium, trypsin, fetal bovine serum (FBS), and phosphate buffer solution (PBS) were purchased from Thermo‐Fisher Scientific Inc. (Shanghai, China). Other chemicals and reagents were used as received.

### 4.2 Ethical Approval and Informed Consent

The animal experiments conducted in this study were approved by the Animal Ethics and Welfare Committee of Hubei University and were carried out in strict accordance with the Guidelines for the Care and Use of Laboratory Animals of Hubei University. The experimental protocols were specifically approved by the Animal Ethics and Welfare Committee of Hubei University (Wuhan, China) under Approval No. 20250003.

### 4.3 Preparation of PMP Nanoparticles

#### 4.3.1 Synthesis of Pt Nanoparticles

Pt nanoparticles were synthesized using a hydrothermal method. Briefly, 50.68 mg of H_2_PtCl_6_·6H_2_O, 222 mg of PVP and 20 mL of ethylene glycol were mixed in a 50 mL two‐neck flask. The precursor solution was reacted with constant stirring at 180°C for 2 h, during which the color of the solution changed from light yellow to black, yielding the PVP‐protected Pt nanoparticles. The reaction mixture was cooled to room temperature. After that, 20 mL of acetone was added to precipitate the Pt nanoparticles. The products were collected by centrifugation at 4500 rpm for 5 min, and washed with acetone and n‐hexane for three times to remove residual PVP. The Pt nanoparticles were vacuum‐dried at 50°C for 6 h, and then dispersed uniformly in methanol by ultrasonication to prepare a black Pt solution with a concentration of 1 mg/mL for further study.

#### 4.3.2 Synthesis of PM Nanoparticles

FeCl_3_ of 0.54 g·6H_2_O and 0.36 g of 2‐aminoterephthalic acid were dissolved in 28 mL of methanol. After that, 2 mL of Pt solution was added dropwise with constant stirring. The mixture was transferred into a 50 mL autoclave and reacted at 100°C for 24 h. After cooling to room temperature, the obtained PM was collected by centrifugation at 5000 rpm for 5 min, followed by washing with acetone and methanol three times. According to the same protocols, neat MIL‐NH_2_ without Pt nanoparticles was also synthesized and coded as MIL.

#### 4.3.3 Synthesis of PMP Nanoparticles

Isopropanol of 100 µL was added to 10 mL of PTA solution, and irradiated under UV light until the color of the mixture changed from colorless to deep blue. The mixture was then blended with 50 mg of PM nanocomposites and stirred for another 2 h. The products (PMP) were collected by centrifugation at 5000 rpm for 5 min, followed by washing with methanol and water three times. PMP nanoparticles were vacuum‐dried at 50°C and stored at room temperature for further study.

### 4.4 Preparation of GP‐n Hydrogels

GelMA with approximately 30% methacrylation was synthesized according to our previous report [58]. PMP nanoparticles were mixed with 15% GelMA solution and photo‐initiator solution by mechanical stirring. The mixture was then centrifuged to remove air bubbles, poured into molds, and crosslinked by UV light. The obtained GelMA/PMP composite hydrogels were coded as GP‐n (n = 0.0.2, 0.5 and 0.8, representing the relative mass fraction of PMP nanoparticles in the hydrogel precursor solution). The compositions and preparation parameters of GP‐n hydrogels are summarized in Table S3.

### 4.5 Preparation of Bilayer GPM Microneedles

In this study, a bilayer microneedle with a base layer of GP‐0 hydrogel and a tip layer of GP‐0.5 hydrogel was designed for the transdermal delivery of PMP nanoparticles. In accordance with a previous report, this microneedle (GPM) was prepared using a mold‐casting method [59]. For the controls, GelMA microneedles (coded as GM) were also prepared using the same protocols.

### 4.6 Physicochemical Characterizations

The morphology and element compositions of the nanoparticles were characterized using a combination of scanning electron microscope (SEM, SIGMA500, Zeiss, Germany) and a transmission electron microscope (TEM, FEI Talos F200x, Thermo‐Fisher, USA). The nanoparticles were further analyzed by Fourier transform infrared spectroscopy (FT‐IR, Nicolet IS‐50, Thermo‐Fisher, USA), x‐ray diffraction (XRD, D8 Advance, Bruker, Germany) and x‐ray photoelectron spectroscopy (XPS, EscaLab 250Xi, Thermo‐Fisher, USA). For FT‐IR analysis, potassium bromide (KBr) pellets were prepared, and the data were collected in the wavenumber range of 400–4000 cm^−1^. XRD analysis was performed using Cu Kα radiation as the monochromatic x‐ray source, with diffraction patterns recorded in the 2θ range of 5° ‐ 70° at a scanning rate of 10°/min. The thermal stability of the nanoparticles was evaluated by thermogravimetric analysis (TG, STA 2500 Regulus, Netzsch, Germany). TGA analysis was conducted under a continuous nitrogen flow with a heating rate of 10°C/min, and weight loss data were recorded over a temperature range of 30 °C – 800°C. Zeta potential was determined by dynamic light scattering (DLS, Zetasizer Pro, Malvern, UK).

### 4.7 Evaluations of Photocatalytic Performances

A UV–vis spectrophotometer (UV–vis, UV3600, SHIMADZU, Japan) was used to measure light absorption efficiency. The photoelectrochemical conversion performances were evaluated using an electrochemical work station (CHI660E, CH Instruments, China). Specifically, the MIL and PMP nanoparticles were uniformly dispersed to prepare a 5 mg/mL solution, which was then spin‐coated onto a 1.5 cm × 1.5 cm fluorine‐doped tin oxide (FTO) conductive glass substrate. After drying, the working electrode was obtained. A three‐electrode system was employed for electrochemical measurements, consisting of an Ag/AgCl electrode as the reference electrode, a platinum (Pt) electrode as the counter electrode, and the working electrode. A 5% sodium sulfate (Na_2_SO_4_) solution served as the electrolyte. According to standard protocols, electrochemical impedance spectroscopy (EIS), linear sweep voltammetry (LSV) and i‐t curves were performed at a bias voltage of 0.5 V to analyze the charge transfer and photoelectrochemical response.

Photoluminescence (PL) spectroscopy was analyzed using a Steady‐state and Transient Fluorescence Spectrometer (FLS1000, Edinburgh, England). The excitation wavelength was set to 365 nm, and fluorescence within the range of 400– 850 nm was collected for data analysis. Time‐resolved photoluminescence (TRPL) decay spectra were recorded under excitation at 375 nm from a xenon lamp, with emission detected at 450 nm. The photocatalytic hydrogen/oxygen evolution performance was also detected. In brief, the PMP nanoparticles were uniformly dispersed to prepare a 5 mg/mL solution, which was then transferred into a 20 mL rubber‐sealed sample vial. High‐purity nitrogen gas was continuously purged to remove residual hydrogen and oxygen. Subsequently, the vial was irradiated with visible light (PLS‐SXE300 xenon lamp with a 400–‐780 nm filter, 50 W power, 50 mm distance, 30 mm spot diameter) to initiate the photocatalytic reactions. A gas‐tight syringe was used to extract the evolved gas. The content of hydrogen and oxygen was measured using a gas chromatograph (7890B, Agilent, USA). Specifically speaking, during the measurements, gas samples were directly collected using gas sampling bags and introduced into the GC system, with an injection volume of 1 mL per sample. For data normalization, the sum of all corrected peak areas was considered as 100%, and the proportion of each component was then calculated accordingly.

### 4.8 DFT Calculation

Density functional theory (DFT) calculation was performed using the Vienna Ab initio Simulation Package (VASP), employing the generalized gradient approximation (GGA) and the Perdew‐Burke‐Ernzerhof (PBE) exchange‐correlation functional. The electron‐ion interactions were described using the projector augmented wave (PAW) potentials, while valence electrons were treated with a plane‐wave basis set, applying a kinetic energy cutoff of 450 eV. DFT‐D3 empirical correction method was incorporated to evaluate the van der Waals interactions. Structural optimizations were carried out until the forces on all atoms were below 0.05 eV/Å. For Brillouin zone integration, a Gamma‐centered (1 × 1 × 1) k‐point mesh was adopted, and spin polarization was included in all calculations.

The free energy changes (ΔG) of each elementary step in the oxygen evolution reaction (OER) and hydrogen evolution reaction (HER) were evaluated using a computational hydrogen electrode (CHE) model. In this approach, the chemical potential of a proton‐electron pair was set to half the energy of a gas‐phase H_2_ molecule at 0 V vs. the reversible hydrogen electrode (RHE). The applied electrode potential (U vs. RHE) was incorporated by adjusting the free energy as G(U) = G (0 V)—neU, where e is the electron charge, n is the number of transferred proton‐electron pairs, and U is the potential. The Gibbs free energy was computed as:

$$\Delta G=\Delta E+\Delta E_{\mathrm{ZPE}}-T\Delta S,$$

where ΔE, ΔE_ZPE_, and ΔS represent the changes in DFT total energy, zero‐point energy, and entropy at 298.15 K, respectively.

### 4.9 Characterizations of the Composite Hydrogels and Microneedles

#### 4.9.1 Morphological Characterization of Hydrogels and Microneedles

The gelation process of the hydrogels was visually recorded using a digital camera before and after gel formation. For microscopic characterization, the freeze‐dried hydrogels were fractured in liquid nitrogen, and the cross‐sections were collected. The morphological features of the hydrogel cross‐sections were then examined using a scanning electron microscope.

#### 4.9.2 Mechanical Property Testing of Hydrogels

The hydrogels were molded into cylindrical specimens (height: 0.5 cm, diameter: 1.5 cm) using a 24‐well plate. Uniaxial compression tests were performed using an universal testing machine at a constant compression rate of 1 mm/min. The deformation behavior during compression was simultaneously recorded using a digital camera for visual analysis.

#### 4.9.3 Swelling Ratio Measurement of Hydrogels

The hydrogels were cut into cubic specimens (0.5 cm × 0.5 cm × 0.5 cm) and vacuum‐dried at 60°C for 24 h to completely remove moisture. The dry weight (W_0_) of each sample was measured using an analytical balance. Subsequently, the dried hydrogels were immersed in PBS buffer solution. At predetermined time intervals, the samples were removed from the solution, and the surface moisture was carefully blotted with filter paper. The swollen weight (W_t_) was then measured using the analytical balance. The water uptake ratio (WUR) was calculated according to the following equation:

$$\mathrm{WUR}=\left(W_{t}-W_{0}\right)/W_{0}\times 100\%$$

#### 4.9.4 Water Contact Angle Measurement of Hydrogels

The hydrogels were cast into thin films using a mold and allowed to dry at room temperature. The static water contact angles of the prepared hydrogel films were measured using a contact angle goniometer. The contact angle values were calculated by the angular measurement method.

#### 4.9.5 Mechanical Performance of Microneedle Tips

The puncture force of the microneedle tips was evaluated using a universal testing machine. The compression distance was set to 600 µm, corresponding to the length of the microneedle tip, and the compression tests were performed at a constant compression rate of 0.1 mm/min.

### 4.10 In Vitro Drug Release Study of Microneedles

Rhodamine B solutions with standard concentrations (1, 3, 5, 25, 50, 75 and 100 mg/mL) were prepared. The absorbance of each solution in the range of 450–‐650 nm was measured using a UV–vis spectrophotometer. The absorbance values at the maximum absorption wavelength of 554 nm were used for linear fitting to establish a concentration‐absorbance standard curve for Rhodamine B. Bilayer microneedles loaded with Rhodamine B were immersed in 5 mL of deionized water and incubated in a 37°C water bath. At predetermined time intervals (1, 2, 4, 6, 12, 24, and 48 h), the immersion solution was completely removed, and 5 mL of fresh deionized water was added. The absorbance of each collected solution in the range of 450–‐650 nm was measured by UV–vis spectrophotometry. The absorbance values at 554 nm were substituted into the linear regression equation to calculate the released concentration of Rhodamine B in the immersion solutions at different time points. The release curve of Rhodamine B from the microneedles over time was then plotted.

### 4.11 Skin Penetration Test of Microneedles

BALB/c‐nu nude mice (4‐week‐old females) were selected for skin penetration experiments due to their hairless phenotype, which facilitates visual observation of microneedle penetration. Prior to testing, the mice were anesthetized safely using isoflurane inhalation. The dorsal skin was surgically excised using sterile instruments and mounted on a flat platform. The microneedles were then applied to the excised skin with a 10 s constant pressure application. Penetration performance was documented and analyzed using digital imaging. After microneedle application, the skin tissues were collected and processed for H&E staining to further assess the penetration status and insertion depth of the microneedles into the skin.

### 4.12 Biocompatibility Evaluations in Vivo

Female Sprague‐Dawley (SD) rats, weighing 180–200 g, were provided by Sai‐Mei Biotechnology Co., Ltd. (Wuhan, China). Before the surgery, all animals were fed in a specific pathogen‐free (SPF) experimental environment for 7 days.

A subcutaneous transplantation model was established. All animals were randomly divided into three groups (n = 5). GM group and GPM group were transplanted with a piece of GM microneedle and GPM microneedle, respectively. B.C. group was sham‐operated. The wound was sutured with absorbable sutures. After the surgery, all animals were conventionally fed for another 15 days.

Fresh whole blood was collected from the animals and immediately detected with the help of the Clinical Laboratory Center of Zhongnan Hospital. The materials and the surrounding capsules, as well as the organs, were harvested for a series of histological analyses. Powerful Biology Co., Ltd. (Wuhan, China) performed H&E staining and immunofluorescence staining. A laser confocal microscope (SpectraPlex, Leica, Germany) was used for data acquisition.

### 4.13 Wound Healing Evaluations

A diabetic rat model was established in Sprague‐Dawley (SD) rats through a combination of high‐sugar, high‐fat diet feeding and intraperitoneal streptozotocin (STZ) administration. The level of blood glucose and body weight were dynamically monitored for 7 days. The model was successfully established until the SD rats exhibited continuous body weight loss and sustained blood glucose levels above 15 mmol/L. These animals were randomly divided into five independent groups (n = 6), including B.C. (Blank Control), 3M Tegaderm3586 Transparent Film Dressing (commercial dressing group), GM+Vis, GPM, and GPM + Vis.

All animals were anesthetized by isoflurane inhalation, and the hair on their backs was completely removed using an electric shaver. A standardized 1.5 cm × 1.5 cm full‐thickness skin defect reaching the deep fascia was created on the dorsal skin using a biopsy punch and a template, without application of an anti‐contraction splint. The wound dressing materials from each group were fixed onto the wound sites. GPM + Vis group was additionally treated with visible light irradiation. All rats were fed for 18 days to allow skin tissue regeneration. The images of wounds were captured every 3 days using a digital camera. For wound area quantification, ImageJ software was used as follows: the scale was first calibrated based on the known ruler dimensions in the image; then the wound margin was manually traced along the wound edge to define a closed loop, and the enclosed area was calculated automatically by the software. Wound closure rate (WCR) at each time point was calculated.

On Day 6 and Day 18, neo‐skin tissues were harvested. A portion of the tissue was immediately frozen in liquid nitrogen for frozen section and RNA sequencing. The others were fixed in a 4% PFA solution for histological analysis. In this study, a series of histological and immunofluorescence staining, such as iNOS, AGEs, CD31, and Ki67, were detected according to standard protocols. The antibodies used in this study are listed in Table S4. The semi‐quantitative analysis of histological images was performed using ImageJ software.

### 4.14 Mechanism Investigations

The gene expression of these neo‐skin tissues was detected by RNA sequencing according to the standard protocols

#### 4.14.1 RNA‐seq

Two sample groups were selected for RNA sequencing analysis, including GPM and GPM+Vis. mRNA was extracted from fresh skin tissue using the KAPA Stranded mRNA‐Seq Kit. RNA fragments with polyA tails were captured using the oligo‐dT beads kit, followed by incubation with the strand synthesis master mix to transcribe mRNA into cDNA. Subsequently, transcriptome sequencing analysis was performed using the Illumina NovaSeq 6000 RNA platform. Based on the standard (*p* value < = 0.05 and |Log2(fold change)| ≥1), FDR ≤ 0.05differential expression gene (DEGs) were evaluated using edge R package. Afterward, used clusterProfiler R package to test the GO analysis, KEGG enrichment analysis. Obtain ferroptosis‐related genes from the Ferrdb database, then ggvenn R package was used for data intersection of RNA‐seq DEGs and ferroptosis‐related genes, the pheatmap R package was used for the display of the heatmap of the intersection genes, used ggplot R package display the bubble map of the intersection genes. Protein‐Protein Interaction Networks was conducted using the String database.

To validate the RNA‐seq findings on ferroptosis‐related gene expression, we performed immunoblotting to assess key ferroptosis‐associated proteins. Proteins were extracted, mixed with 1× Laemmli loading buffer, and separated by SDS‐PAGE. After transfer to a methanol‐activated PVDF membrane (Millipore), blocking was conducted with 5% nonfat milk in TBST. Membranes were probed with primary antibodies (diluted in 3% BSA) overnight at 4°C, followed by HRP‐conjugated secondary antibodies. Protein bands were visualized using FDbio‐Pico ECL and imaged with a chemiluminescence detection system.

To examine mitochondrial morphological changes indicative of ferroptosis suppression, cells were processed for TEM (HT7700, Hitachi, Japan). Samples were fixed in 2.5% glutaraldehyde, postfixed with 1% osmium tetroxide, and stained with uranyl acetate. After dehydration in an ethanol gradient, samples were embedded in resin, polymerized, and ultrathin‐sectioned using a Leica Ultramicrotome. Sections were stained with uranyl acetate/lead citrate and imaged under 80 kV accelerating voltage to assess mitochondrial cristae structure and membrane integrity. Additionally, lipid peroxidation levels were evaluated by measuring the intracellular contents of malondialdehyde (MDA) and glutathione (GSH) using commercial assay kits.

#### 4.14.2 Western Blotting

Total cellular protein was isolated in Radio Immunoprecipitation Assay (RIPA) lysis buffer (Servicebio, China) supplemented with a protease inhibitor cocktail (MCE, China) and PMSF (Servicebio, China). Protein concentrations were determined using a BCA protein assay kit (HYCEZMBIO, China). Equal amounts of protein were resolved by SDS‐PAGE and then transferred onto polyvinylidene fluoride (PVDF) membranes (Millipore). Membranes were blocked with 5% non‐fat milk at room temperature for 1 h, followed by three washes in Tris‐buffered saline containing 0.1% Tween‐20 (TBST) for 10 min each. Subsequently, membranes were incubated with the indicated primary antibodies overnight at 4°C. The next day, after incubation with the appropriate horseradish peroxidase (HRP)‐conjugated secondary antibodies for 1 h at room temperature, protein bands were visualized using an ECL substrate kit (HYCEZMBIO, China), and chemiluminescent signals were captured with an Alliance Q9 imaging system (UVITEC, England). Antibodies used included primary antibodies against TNFAIP3, FTH1, β‐Actin, PTGS2, NFE2L2, and hnRNPA1.

#### 4.14.3 RNA Preparation and qRT‐PCR

Total RNA from cell lines was isolated using TRIzol reagent (Invitrogen, USA) according to the manufacturer's protocol. The extracted RNA was reverse transcribed into cDNA with HiScript III RT SuperMix for qPCR (Vazyme, China). Quantitative real‐time PCR was then performed using SYBR Green Master Mix (Vazyme, China) on a StepOnePlus Real‐Time PCR System (Applied Biosystems, USA), and relative transcript levels were calculated using the 2^−ΔΔCt method.

#### 4.14.4 Co‐Immunoprecipitation (Co‐IP)

Cells were lysed on ice for 1 h in 2 mL NP‐40 lysis buffer (Beyotime, China) supplemented with a protease inhibitor cocktail (MCE, China) and PMSF (Servicebio, China). Five percent of the lysate was reserved as the input control, and the remaining lysate was divided equally into two fractions, which were incubated overnight at 4°C with 4 µg of primary antibody or control IgG, respectively. Protein A/G magnetic beads (MCE, China) were then added to each fraction and incubated for 3 h at 4°C. After five washes, the immunoprecipitated complexes bound to the beads were collected and subsequently analyzed by western blotting or mass spectrometry. The antibodies used are described in the western blotting section.

#### 4.14.5 Silver Staining and Mass Spectrometry Analysis

Following completion of electrophoresis, 7.5% SDS‐PAGE gels were silver‐stained using the PAGE Gel Silver Staining Kit (Beyotime, China). Mass spectrometry analysis was subsequently carried out by Shanghai Bioprofile Technology Co., Ltd. Differential protein identification and quantification were performed using Proteome Discoverer software (version 1.4, Thermo Fisher Scientific).

#### 4.14.6 Ubiquitination Assays

For ubiquitination analysis, L929 cells (TNFAIP3‐WT, TNFAIP3‐KD and TNFAIP3‐OE) were transfected with HA‐ubiquitin plasmids. After 24 h, cells were treated with 10 µM MG132 (MCE, China) for an additional 24 h, followed by Co‐IP as described above. The ubiquitination of hnRNPA1 in the immunoprecipitates was assessed by western blotting, and β‐actin levels were examined in whole‐cell lysates as a loading control.

### 4.15 Statistical Analysis

Statistical analysis was performed using GraphPad Prism 11. Data normality (Shapiro‐Wilk) and variance homogeneity (Levene's) were checked. One‐way analysis of variance (ANOVA) followed by Tukey's honestly significant difference (HSD) post hoc test were employed. Quantitative data are expressed as mean ± standard deviation (SD). At least three independent samples were included. All tests were two‐sided, with significance set at *p* < 0.05.

## Author Contributions

Conceptualization: Z.C., Z.W. and B.L. Methodology: Z.C., Y.Y., X.X. and Z.W. Investigation: W.W., W.C., Y.Y. and Z.Z. Data curation: Z.C., Y.Y., Z.Z. and Y.Y. Formal analysis: J.L., X.G. and H.Y. Visualization: Z.C. and Z.W. Writing – original draft: Z.C., Y.Y. and X.X. Writing – review and editing: Z.W., Y.C., K.C. and B.L. Resources: B.L., X.X. and Z.W. Validation: Z.W., B.L. and K.C. Supervision: Z.W., Y.C., and B.L. Project administration: Z.W., B.L. and X.W. Funding acquisition: B.L., X.X., Z.W., Y.C. and W.H.

## Conflicts of Interest

The authors declare no conflicts of interest.

## Supporting information




**Supporting File**: advs77413‐sup‐0001‐SuppMat.docx.

## Data Availability

The datasets generated and/or analysed during the current study are publicly available in Mendeley Data, https://data.mendeley.com/datasets/5xbxx5bj6c/1.
