# Supplementary material for: Integrated O2 and H2 Gas Therapy via Microneedle‐Assisted Photocatalytic Water Splitting for Accelerating Diabetic Full‐Thickness Skin Wound Healing
Source: Adv Sci (Weinh). 2026 Aug 29:e77413. Online ahead of print. doi: 10.1002/advs.77413 (PMC13525420; doi:10.1002/advs.77413)
Supplement: Supplementary file 1 [file ADVS-9999-e77413-s001.docx]

**Supporting Information**

**Integrated O_2_ and H_2_ Gas Therapy via Microneedle‑Assisted Photocatalytic Water Splitting for Accelerating Diabetic Full-Thickness Skin Wound Healing**

Zesheng Chen^1,2,†^, Yanchao Yu^1,†^, Xingyuan Xiao^1,†^, Wenwen Cheng^3^, Ye Yang^3^, Zhengyao Zhang^2,4^, Wang Wang^1^, Zhijun Zhou^1^, Weikang Hu^3^, Xiaolong Wang^5^, Bing Li^1^🖂, Kwang Leong Choy^4^🖂, Yun Chen^2^🖂 & Zijian Wang^1,2^🖂

^1^ Department of Urology, Hubei Key Laboratory of Urological Diseases, Institute of Urology, Zhongnan Hospital of Wuhan University, Wuhan 430071, China

^2^ Department of Biomedical Engineering, TaiKang Medical School (School of Basic Medical Sciences), Wuhan University, Wuhan 430071, China

^3^ School of Materials Science and Engineering, Stem Cells and Tissue Engineering Manufacture Center, Hubei University, Wuhan 430062, China

^4^ Suzhou Key Laboratory of Advanced Sustainable Materials & Technologies, Division of Natural and Applied Sciences, Duke Kunshan University, Kunshan 215316, China

^5^ Department of Pathology and Laboratory Medicine, Temple University Lewis Katz School of Medicine, Philadelphia, Pennsylvania, USA

^†^ These authors contributed equally: Zesheng Chen, Yanchao Yu, Xingyuan Xiao

🖂e-mail: [zijianwang@whu.edu.cn](mailto:zijianwang@whu.edu.cn); [yunchen@whu.edu.cn](mailto:yunchen@whu.edu.cn); [kwang.choy@duke.edu](mailto:kwang.choy@duke.edu); [bingli2023@whu.edu.cn](mailto:bingli2023@whu.edu.cn)

**Results**

**Figure S1.** Length distribution plots for MIL (A), PM (B) and PMP (C) nanoparticles.

**Figure S2.** Standard XRD patterns of MIL nanoparticles, PTA and Pt nanoparticles.

**Figure S3.** The FT-IR spectrum of MIL, PMP and PTA.

**Figure S4**. XPS spectrum of Fe 2p (A), C 1s (B) and O 1s (C) signals for PMP nanoparticles signals.

**Figure S5**. The adsorption and desorption curves of MIL (A) and PMP (B) nanoparticles.

**Figure S6**. The BET specific surface area of MIL and PMP nanoparticles.

**Figure S7.** The bandgap of MIL (A) and PMP (B) nanoparticles.

**Figure S8.** The VB-XPS spectra of MIL (A) and PMP (B) nanoparticles.

**Figure S9.** The linear voltammogram curves (A) and i-t curve (B) of MIL and PMP nanoparticles.

**Figure S10.** TRPL spectra of MIL and PMP nanoparticles.

**Figure S11.** The compressive mechanical curve of microneedles.

**Figure S12.** H&E‑stained images of skin after GPM microneedle puncture. Scale bar: 100 μm.

**Figure S13.** The drug releasing capability of GPM was evaluated using an improved Rhodamine B (Rh. B)-releasing model. (A) Absorbance standard curve of Rh. B in PBS at 550 nm at various concentrations; (B) The Rh. B-releasing curves of GM and GRM (n = 3). Values are expressed as the mean ± SD.

**Figure S14.** Immunofluorescence staining (A) and quantitative analysis (B) of CD206 in fibrous capsules (n = 3). Scale bar: 75 μm. All data are presented as the mean ± SD. *n.s.* indicates no significance.

**Figure S15.** Blood biochemical results (n = 3). Total protein (TP); albumin (ALB); alkaline phosphatase (AKP); potassium ion (K^+^); sodium ion (Na^+^) and Calcium ion (Ca^2+^). All data are presented as the mean ± SD. *n.s.* indicates no significance.

**Figure S16.** Changes in body weight (A, n = 30) and blood glucose levels (B, n = 5). All data are presented as the mean ± SD.

**Figure S17.** Immunohistochemistry (IHC) images (A) and quantitative results (B-C, n = 3) of Col-I and Col-III at Day 18. Scale bar: 100 μm. All data are presented as the mean ± SD. ***P* < 0.01, ****P* < 0.001.

**Figure S18.** Immunofluorescence (IF) images and the quantitative results (n = 3) of GPX4 at Day 6. Scale bar: 50 μm. All data are presented as the mean ± SD. *n.s.* indicates no significance, **P* < 0.05, ****P* < 0.001.

**Figure S19.** The relative contents of MDA (A) and GSH (B) (n = 3). All data are presented as the mean ± SD. **P* < 0.05, ***P* < 0.01, ****P* < 0.001.

**Table S1.** Gibbs free energy of elementary reactions in HER

| HER | MIL | PMP |
| --- | --- | --- |
| H^+^+e→H^*^ | 1.464 | -0.300 |
| (H^+^+e) +H^*^→H_2_ | -1.464 | 0.3 |

**Table S2**. Gibbs free energy of elementary reactions in OER

| OER | MIL | PMP |
| --- | --- | --- |
| OH^-^+*→OH^*^+e^-^ | 1.395 | 1.649 |
| OH^*^+ OH^-^→ O^*^+ H_2_O + e^-^ | 1.863 | 1.487 |
| O^*^+ OH^-^→ OOH^*^+ e^-^ | 0.980 | 1.475 |
| OOH^*^+ OH^-^→ O_2_+ H_2_O + e^-^ | 0.682 | 0.309 |

**Table S3.** The codes, compositions and preparation parameters of GP-n hydrogels.

|  | 15% GelMA  (mL) | PMP  (mg) | 10% 2959  (μL) | UV power  (W) | Curing time  (s) |
| --- | --- | --- | --- | --- | --- |
| GP-0 | 20 | 0 | 200 | 500 | 300 |
| GP-0.2 | 20 | 6 | 200 | 500 | 300 |
| GP-0.5 | 20 | 15 | 200 | 500 | 300 |
| GP-0.8 | 20 | 24 | 200 | 500 | 300 |

**Table S4.** List of primary antibodies and secondary antibodies

| Name | Species | Dilution | Resources |
| --- | --- | --- | --- |
| CD45 | Rabbit | 1:500 | Proteintech, China, Cat No. 20103-1-AP |
| CD80 | Mouse | 1:1000 | Proteintech, China, Cat No. 66406-1-Ig |
| HIF-1α | Rabbit | 1:1000 | Cell Signaling Technology, USA, cat. #36169 |
| iNOS | Rabbit | 1:800 | Cell Signaling Technology, USA, cat. #13120 |
| CD206 | Rabbit | 1:500 | Proteintech, China, Cat No. 18704-1-AP |
| CD31 | Rabbit | 1:500 | Proteintech, China, Cat No. 11265-1-AP |
| Ki67 | Rabbit | 1:500 | Proteintech, China, Cat No. 11265-1-AP |
| Col-I | Mouse | 1:10000 | Proteintech, China, Cat No. 67288-1-Ig |
| Col-III | Rabbit | 1:1000 | Proteintech, China, Cat No. 22734-1-AP |
| GPX4 | Rabbit | 1:500 | Proteintech, China, Cat No. 30388-1-AP |
| TNFAIP3 | Mouse | 1:50000 | Proteintech, China, Cat No. 66695-1-Ig |
| SCL7A11 | Rabbit | 1:1000 | Proteintech, China, Cat No. 32384-1-AP |
| GAPDH | Mouse | 1:50000 | Proteintech, China, Cat No. 60004-1-Ig |
| PTGS2 | Rabbit | 1:4000 | Proteintech, China, Cat No. 27308-1-AP |
| FTH1 | Rabbit | 1:5000 | Proteintech, China, Cat No. 11682-1-AP |
| NFE2L2 | Rabbit | 1:5000 | Proteintech, China, Cat No. 16396-1-AP |
| hnRNPA1 | Rabbit | 1:5000 | Proteintech, China, Cat No. 11176-1-AP |
| Actin | Mouse | 1:100000 | Proteintech, China, Cat No. 66009-1-Ig |
| HA | Rabbit | 1:10000 | Proteintech, China, Cat No. 51064-2-AP |
| Anti-Mouse-IgG (H+L)- CoraLite®488 | Goat | 1:1000 | Proteintech, China, Cat No. SA00013-1 |
| Anti-Rabbit-IgG(H+L)- CoraLite®488 | Goat | 1:1000 | Proteintech, China, Cat No. SA00013-2 |
| Anti-Mouse-IgG (H+L)- CoraLite®594 | Goat | 1:500 | Proteintech, China, Cat No. SA00013-3 |
| Anti-Rabbit-IgG(H+L)- CoraLite®594 | Goat | 1:10000 | Proteintech, China, Cat No. SA00013-8 |
| Anti-Mouse-IgG (H+L)-HRP | Goat | 1:10000 | Proteintech, China, Cat No. SA00001-1 |
| Anti-Rabbit-IgG(H+L)-HRP | Goat | 1:10000 | Proteintech, China, Cat No. SA00001-2 |
